# The influence of Cajal and Lorente de Nó on Donald O. Hebb’s neuropsychological theory

**DOI:** 10.3389/fnana.2026.1861011

**Published:** 2026-07-27

**Authors:** Richard E. Brown

**Affiliations:** Department of Psychology and Neuroscience, Dalhousie University, Halifax, NS, Canada

**Keywords:** cell assemblies, Hebb synapses, history, reverberating circuit, unpublished notes and papers

## Abstract

Donald O. Hebb was born in Chester Nova Scotia on 22 July 1904, attended the Chester school and the Halifax County Academy, and obtained a BA in English and Philosophy from Dalhousie University in 1925. After obtaining a teacher’s certificate he taught in his old school in Chester for a year and then in Montreal while attending McGill University to obtain his MA in Psychology as a part-time student. He moved to Chicago to work with Karl Lashley and then completed his PhD with Lashley at Harvard in 1936. He was an instructor for a year at Harvard (1936–37) and then received a Fellowship to work at the Montreal Neurological Institute (1937–39), conducting neuropsychological tests on the patients of Dr. Wilder Penfield. From 1939 to 1942 he was a professor at Queen’s University in Kingston, Ontario and then a Research Associate of Professor Lashley, studying emotionality in chimpanzees at the Yerkes Primate Center in Florida (1942–1947). It was there that he began to write his influential book, *The Organization of Behavior*. This book introduced the concepts of synaptic change, cell assemblies, and phase sequences to account for the neural events underlying behavior. Hebb’s work revolutionized the study of brain and behavior by establishing a biological basis for psychological phenomena. Hebb became a professor of Psychology at McGill University in 1947 and head of this department in 1948, completing his book at McGill in 1949. The crucial element in Hebb’s theory was the concept of reverberatory circuits of Lorente de Nó, based on Cajal’s concept of closed circuits. This paper examines the unpublished notes and papers written by Hebb between 1932 and 1947 as he developed his theory of neuropsychological function based on the concept of closed or reverberatory circuits and how this theory was used in psychology, neuroscience and the computer modeling of brain functions.

## 1 Introduction

Donald O. Hebb developed his cell assembly theory of neural function underlying behavior over at least 17 years, starting with his MA thesis at McGill University in 1932 and culminating in the publication of *The Organization of Behavior* in 1949 (Brown, 2020). There were many steps along the way, but the most important was Hebb’s application of “Lorente de Nó circuits” to his neuropsychological theory of perception and memory as these explained how a stimulus could be retained in the brain circuitry after its termination. This paper briefly summarizes Hebb’s education and academic career and then focuses on the influence of Cajal and Lorente de Nó on the development of Hebb’s neuropsychological theory. To follow this development, I have used a number of unpublished notes and papers written by Hebb between 1932 and 1947 which show the evolution of his thoughts about the “Hebb synapse,” the cell assembly and phase sequence. Portions of these unpublished papers, which are held in the McGill University Archives, are attached as supplemental files so that they are accessible to readers of this paper.

## 2 Hebb’s education and academic career

Donald Olding Hebb was born in the village of Chester, Nova Scotia on 22 July 1904, the first of four children. Both of his parents were physicians, and he attended the local Chester School until the tenth grade when the family moved to Dartmouth, Nova Scotia. Hebb completed high school at the Halifax County Academy in Halifax, Nova Scotia, and in 1921, he entered Dalhousie University, majoring in English and Philosophy, with the intention of becoming a novelist (Hebb, 1980a). At that time, Psychology was taught in the Philosophy Department at Dalhousie and Hebb was exposed to William James’s *Textbook of Psychology* and Langfield and Allport’s *Elementary Laboratory Course in Psychology*. After graduating with his BA in 1925, he took summer classes to obtain a teaching certificate from the Provincial Normal School in Truro, Nova Scotia, and became the principal of his old school in Chester for a year (Brown, 2006, 2007; Brown and Milner, 2003).

Hebb then moved to Montreal, where he found a teaching job at Verdun High School (1927–28) and met Professor W. D. Tait, the Head of the Psychology Department at McGill University. In 1928, Hebb became a part-time graduate student in Psychology at McGill under the supervision of Professor Chester Kellogg, while by day he taught at Rushbrooke School in the Montreal suburb of Verdun, Quebec. During the 1930–31 school year, Hebb was bedridden with tuberculosis of the hip, which left him with a permanent limp. During this time, he convalesced at his father’s home in Dartmouth, Nova Scotia, and while he was bedridden, he studied Sherrington (1906) *Integrative activity of the nervous system* and Pavlov (1928) *Lectures on conditioned reflexes* and wrote an MA thesis (Hebb, 1932) which contains his first thoughts on the nature of synaptic activity during classical conditioning (Brown and Milner, 2003). After the completion of his MA thesis, Hebb worked in the laboratory of Boris Babkin, a student of Pavlov, studying Pavlovian conditioning with Leonid Andreyev, who had also come from Pavlov’s laboratory to pursue his research at McGill. However, he became disillusioned with Pavlovian conditioning procedures and his graduate studies at McGill and decided to complete his PhD with Karl Lashley (Hebb, 1959a, 1980a).

In July 1934 Hebb began to work with Lashley at the University of Chicago where he met other students who became life-long friends, including Frank Beach and David Krech (Dewsbury, 2002). While in Chicago, Hebb revised his MA thesis into a paper entitled “The interpretation of experimental data on neural action.” Although never published, this paper (Hebb, 1934) was the second step in his thoughts about how the brain functioned. As a graduate student at McGill and Chicago, Hebb (1933/34) also wrote another unpublished manuscript on *Scientific method in psychology.* Only a year after Hebb arrived in Chicago, however, Lashley accepted a position at Harvard University and Hebb moved to Cambridge, Massachusetts, receiving his PhD from Harvard in 1936 for his thesis work on the visual abilities of rats reared in the dark (Hebb, 1936). For the next year, Hebb worked as a Research Assistant for Lashley and as an Instructor in Introductory Psychology for Professor E. G. Boring. During that year, Hebb published his PhD research (Hebb, 1937a; Hebb, 1937b; Hebb, 1938b) and completed the research that he had started in Chicago on field orientation in rats (Hebb, 1938b; Hebb, 1938c).

In 1937, Hebb was appointed as a Rockefeller Fellow and Lecturer in Clinical Psychology at the Montreal Neurological Institute (MNI), where he gave psychological tests to the temporal lobe and frontal lobe surgery patients of Wilder Penfield (Hebb, 1939a, 1939b). Hebb’s most complete study was of patient KM, who was tested before and after a frontal lobotomy (Hebb and Penfield, 1940). Hebb’s experience in testing patients at the MNI led him to develop a theory about the nature of intelligence and how it should be tested (Hebb, 1942; Hebb and Morton, 1944). Along with professor N. W. Morton of the McGill Psychology Department, Hebb began to develop two new tests for measuring adult intelligence: the verbal *Adult Comprehension Test* and the non-verbal *Picture Anomaly Test* (Hebb and Morton, 1943). Hebb had observed that lesions of different brain areas produced different cognitive impairments and began to believe that intelligence was not a unitary entity but a complex phenomenon, whose multiple components could be differentially affected by cerebral lesions. He therefore concluded that, rather than measuring overall intellectual change, one should determine the specific aspects of intelligence affected by brain lesions using specific neurological tests (Hebb, 1939b). Hebb also came to believe that intelligence had two components; a fixed or innate component and a variable component that could be influenced by environmental experience (Hebb, 1942), a concept that was incorporated by Cattell (1943) into his theory of fluid and crystallized intelligence (see Brown, 2016). Hebb also believed that the age at which a brain injury occurred was important in determining its effects on intelligence; certain abilities were affected more by early than late brain injury, while the age at injury was not important for other abilities (Hebb, 1939b, 1942). These studies were important for the development of Hebb’s neuropsychological theory in *The Organization of Behavior*.

When his Rockefeller Fellowship ended in 1939, Hebb became a Lecturer in Experimental Psychology in the Department of Mental and Moral Philosophy at Queen’s University in Kingston, Ontario. Despite his heavy teaching load at Queen’s, Hebb managed to publish work done at the MNI on the effects of brain lesions at different ages (Hebb, 1942) and a review of frontal lobe function (Hebb, 1945a). He also designed a variable path maze and this Hebb and Williams (1946) has since been used in a plethora of studies of learning in animals and humans (Boutet et al., 2018; Shore et al., 2001).

In 1942, Lashley became the director of the Yale Primate Laboratories at Orange Park, Florida and hired Hebb as a Research Associate (1942–45) and then as a Harvard University Research Fellow (1945–1947) after Harvard took over the primate center. In Florida, Hebb completed studies on emotionality in chimpanzees (Hebb, 1945b, 1949b) and related these findings to human emotionality (Hebb, 1946a, 1946b; Hebb, 1947a). In addition to the primate research, Hebb continued his work on the development of rat intelligence. To determine the effects of early experience on learning, Hebb reared rats as pets at home and showed that enriched experience during development resulted in improved maze learning in adulthood. Although these results were only published as an abstract at an APA meeting (Hebb, 1947b), they formed the basis of studies on the effects of environmental enrichment on behavior and neural development (Krech et al., 1962).

During his years in Florida, Hebb began to think seriously about his theory of brain function and outlined his ideas in a series of unpublished notes (Hebb, 1944a, 1944b; Hebb, 1945c, 1945d). In his paper on the nature of fear, Hebb (1946b) published his first thoughts on the phase sequence as the anatomical bases of “the train of thought and perception” and his first reference to the work of Lorente de Nó (1939). In 1946, Hebb (1946c) completed the first five chapters of the manuscript of a book, eventually published in 1949 under the title *The Organization of Behavior*, in which he outlined a new way of understanding behavior in terms of brain function. In the summer of 1947, Hebb taught summer courses at Harvard University (Rosenzweig, 1998) on his way to Montreal to take up an academic position in Psychology at McGill University where he spent the rest of his career (Brown, 2006, 2007). When he retired in 1977, he moved back to Nova Scotia, becoming a Professor Emeritus at Dalhousie University until his death in 1985. It was here that he wrote his second autobiographical memoir (Hebb, 1980a) and his book *Essay on Mind* (Hebb, 1980b).

## 3 Writing the Organization of Behavior in Florida (1944–47) and Montreal (1947–49)

Hebb discussed how he came to write *The Organization of Behavior* in his two autobiographical memoirs (Hebb, 1959a, 1980a). In short, his previous research on the effects of brain lesions at different ages on intelligence (Hebb, 1942) led him to the conclusion that intelligence must be a product of innate ability and learning experience. He began to ask how the concepts, modes of thought, and ways of perceiving, which constitute intelligence, could be conceived of in terms of neural mechanisms (Hebb, 1980b, p. 292). Hebb wrote the first part of the book in Orange Park, Florida between 1944 and 1946 and then sent copies of these chapters to his colleagues for their comments. He completed his book at McGill between 1947 and 1949, when it was published (see Brown, 2020).

In *The Organization of Behavior*, Hebb (1949a) proposed a theory for the explanation of psychological concepts such as attention, perception and learning in terms of a set of neurophysiological postulates. He proposed the concepts of the “Hebb synapse,” cell assembly, and phase sequence, which have since become central tenants in neuroscience (Cooper, 2005; Eichenbaum, 2018; Kolb, 2003; Spatz, 1996). In his theory, Hebb devised a way to integrate the ideas of the most prominent psychologists of the time (Lashley, Köhler, Tolman and Hull) through common neurophysiological processes. On the basis of this theory, Hebb reinterpreted his previous research on Penfield’s neurosurgery patients, the development of intelligence, animal models of intelligence, and emotionality in chimpanzees.

The first half of the book focused on providing a neural explanation for perception and learning, the attempts of Köhler and Lashley to provide explanations for these processes in terms of field theory and equipotentiality, and then Hebb’s concepts of synaptic plasticity, the cell assembly and phase sequence. These were the five chapters completed in Florida. The second half of the book, completed in Montreal, was on the development of learning capacity and the neural processes related to learning, motivation, emotion, and the growth and decline of intelligence. These were the chapters in which Hebb reinterpreted his previous research. The idea of synaptic change (plasticity) underlying learning was not new and Hebb pointed out that many others had proposed the idea of the “Hebb synapse” before him (see Brown et al. 2021). The long process that Hebb went through to write *The Organization of Behavior* took 17 years (Brown, 2020) and involved many unpublished notes and drafts.

Cooper (2005, page 859) pointed out that: “It is difficult to reconstruct the writing of Hebb’s book, and the genesis of ideas contained within it, but Hebb (1980a) autobiography provides some indications” (see Cooper, 2005, pages 856–857). While this is true if only published materials are consulted, Hebb left a number of unpublished notes, papers and letters (housed in the McGill University Archives in Montreal, Canada, boxes MG1045), which provide considerable insight into the process that he went through in developing his theory. The purpose of this paper is to examine the development of Hebb’s ideas in these unpublished notes and papers, with a focus on the importance of the reverberatory loops of Cajal and Lorente de Nó in the development of his theory, a topic that has been discussed previously (see Brown et al. 2021; Cooper, 2005; de Castro, 2019; Espinosa-Sanchez et al., 2020, 2025; Haider, 2008; Martinez and Gil, 2003; Rodrigues et al., 2020; Tegner et al., 2002) but the current paper is the first to use Hebb’s unpublished notes to show the steps involved in the development of his theory between 1932 and 1949.

## 4 The early development of Hebb’s ideas on the neural basis of behavior: 1932–34

**Figure 1:**
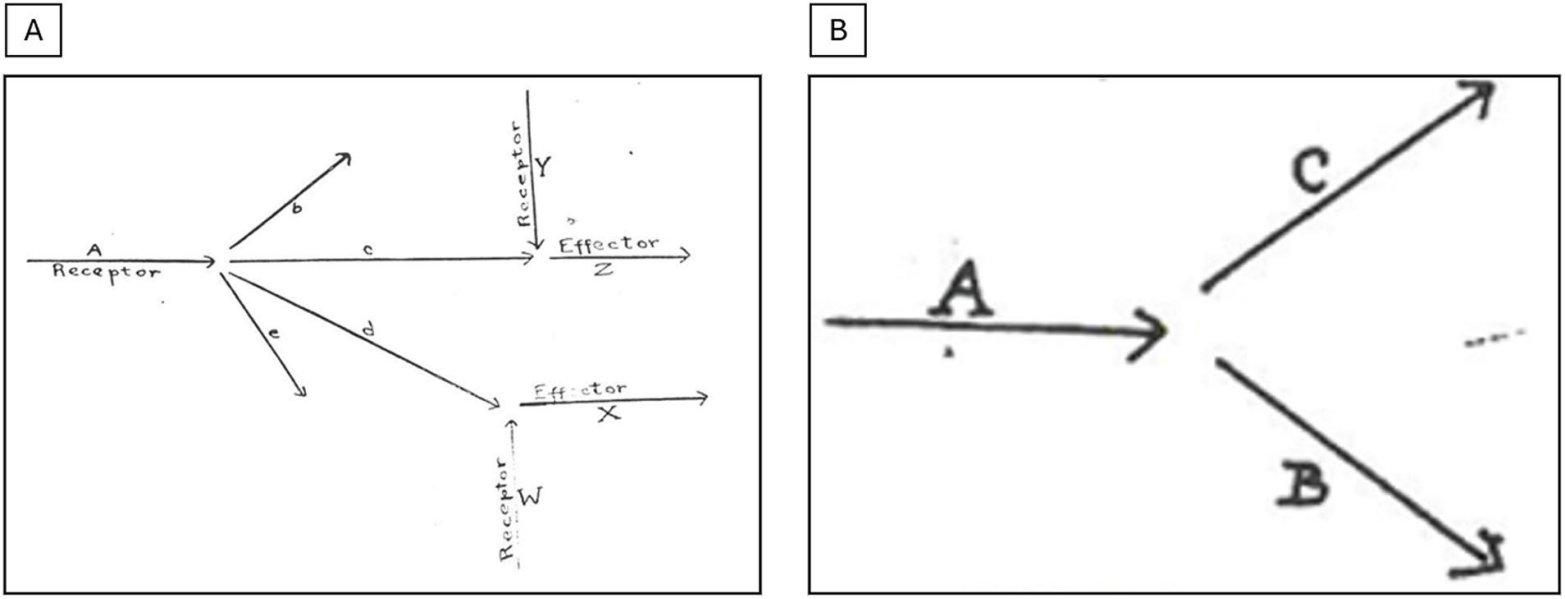
**(A)** Hebb (1932, page 8) explained this figure [his Figure 1, page 9] as follows: In order to understand the functioning of the single neuron in this process [the conditioned reflex] let us take an imaginary nervous organism or part of a nervous organism with three exteroceptors, A, Y, and W, and two effectors, Z and X, making reflex arcs Y-Z and W-X. Other neurons are denoted by b, c, d, and e. Excitation of A by an external agent is to be made a conditioned stimulus for a reaction of Z, and for this purpose the excitation of A is repeatedly followed by excitation of Y, and therefore by a (reflex) discharge of Z. Eventually this will establish a neural route A-c-Z. If the excitation of A is instead followed by the reflex W-X, we know that this will establish a route A-d-X instead of A-c-Z. **(B)** Hebb (1932, page 30; Figure 5) used this figure to explain his concept of unconditioned inhibition as follows: Suppose that in this figure the synaptic resistance between the neuron A (afferent) and each of B and C (efferent) is the same, and that B and C are alternatively active. If A is excited when B is active, more impulses from A will go to B than to C; when C becomes active, and B inactive, the number of impulses to C will increase, and to B decrease. In that case, observing that A had not much tendency to excite C while B was active, or to excite B while C was active, we might say that the activity of B inhibited C, and vice versa. This is basically Sherrington’s concept of reciprocal inhibition.

**Figure 2:**
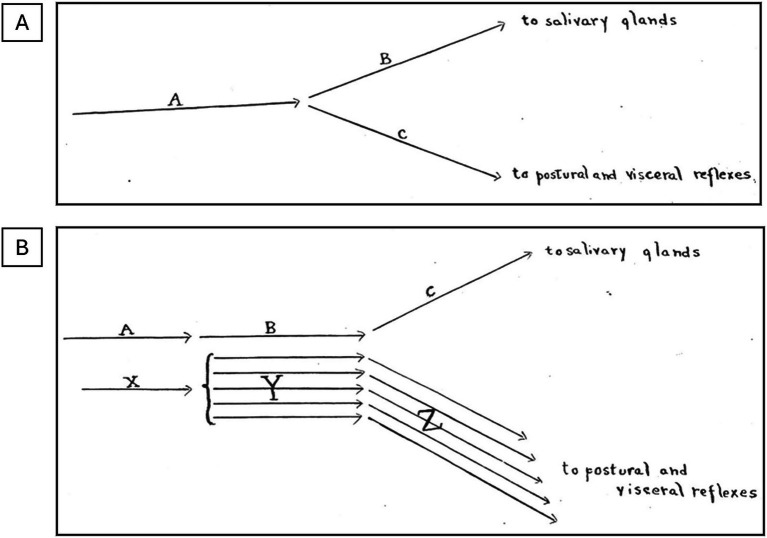
**(A)** In chapter 4 on conditioned inhibition, Hebb (1932, page 47; Figure 8, page 50) gave his conception of Pavlov’s (1928, page 206) first and second types of ‘internal inhibition’ as follows: In this figure A-B represents a route to the salivary reflex, A-C to the continuously active reflexes. To establish a route A-B must have been to establish another A-C as well: for just as activity of the salivary reflex accompanied excitation of A, so did activity of the postural and visceral reflexes. If A is now repeatedly excited, without reinforcing the activity of B, while that of C is reinforced, the activity of C becomes greater than that of B. This increases the number of impulses from A which go to C and decreases the number going to B. Frequent repetition of this would result (by summation) in all impulses from A going to C instead of to B, so that the route A-B temporarily disappears, and the salivary conditioned reflex is inhibited. This is Pavlov’s first internal inhibition. After a short time the conditioned reflex (route A-B) re-appears. This would occur with the disappearance of the temporary facilitation, which was due to the stronger activity of C: the permanent facilitation, or relative synaptic block, would remain practically as before. Repeating the stimulus often enough without reinforcing the route A-B would of course change the synaptic block permanently. In establishing the inhibition of delay (Pavlov’s second type of internal inhibition), the route A-B is established and therefore route A-C as well. After the conditioned reflex is established (i.e., route A-B), excitation of A is followed by the unconditioned salivary stimulus at an interval of 30 s to 3 min. During this interval, that is, the route A-B is un-reinforced, while route A-C is continuously reinforced by the constant reflex activity of the organism. Just as in the extinction of the conditioned reflex, *bahnung* [facilitation] due to the greater activity of C may completely interrupt the route A-B in favor of the route A-C. **(B)** Hebb (1932, page 51–52; Figure 9) gave his conception of Pavlov’s (1928, pages 207–208) third and fourth types of ‘internal inhibition’ [conditioned and differential Inhibition] as follows: The third and fourth types of internal inhibition may almost be considered the same. The third is the inhibition which differentiates two stimuli which are much alike, the fourth conditioned inhibition proper in which an inhibitory stimulus accompanying a conditioned stimulus prevents the conditioned reaction. The inhibition in both third and fourth types is closely parallel to that of the first two. A-B-C is a conditioned route to the salivary glands: stimulation of A has been accompanied by activity of the salivary effectors, C, and also by the effectors of the reflexes of posture, breathing, etc., Z, so that routes A-B-C and A-B-Z are formed. In order to make excitation of X an inhibitory stimulus, it has been repeatedly excited (at the same time as A) without the unconditioned salivary stimulus. This forms a route X-Y-Z. Thus, when A and X are stimulated together repeatedly, reinforcing the activity of Z but not that of C, the stronger activity of Y and Z deflects impulses from the route A-B-C, so that C does not react. When A is stimulated by itself, Y is inactive (being excited only by X) and Z therefore less active, so that the route A-B-C functions as well as A-B-Z.

**Figure 3:**
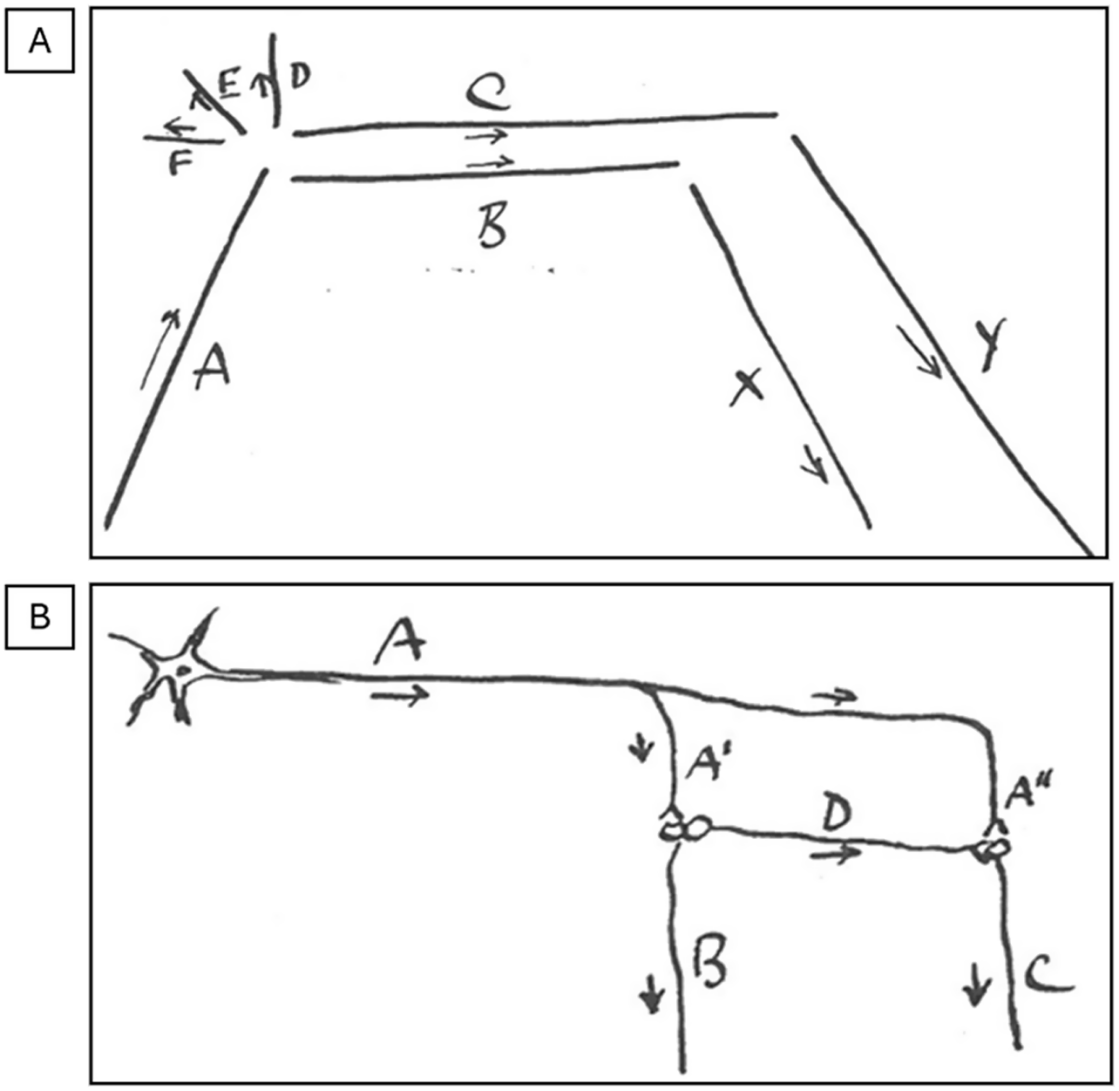
**(A)** Hebb (1934, page 10; Figure 1) used this diagram (Figure 5A) to illustrate his discussion of conditioning. Arrows indicate the direction of transmission of impulses. In this figure, “A represents an afferent tract excited by the stimulus to be conditioned, and X and Y efferent tracts leading, respectively, to the alimentary reflex activity and to some other less active reflexes, the coincidence of excitation in A and reflex activity in X would lead to the formation of a route A-B-X and to the discontinuance of routes A-C-Y, A-D, A-E, etc. This discontinuance must be postulated because presumably the excitation from A being non-specific to any one reaction is spread over the cells which synapse here with A, and since Pavlov’s work apparently shows the disappearance of the first non-specific general reactions to the stimulus. Even a defense-reaction complex will be discontinued (Pavlov, 1927, p. 30), all disturbances of breathing and heart rate disappearing, so that if we accept the conception of specific routes via the cortex we must conclude that the formation of route A-B is accompanied by a discontinuance of A-C, A-D, and so on.” According to Hebb (1934, page 11), this postulate appears to account also for reciprocal innervation. **(B)** Hebb (1934, page 11; Figure 2) gives a second version of his concept of conditioned inhibition (Figure 5B). This shows a representation of a cell which activates two others in the cord. Cell D forms a possible route A-A’-D-C so that impulses in A’ do not necessarily activate B. Arrows show direction of transmission of impulses. His explanation of this Figure (p. 11) is as follows: “The validity of the conception of specific routes in the cortex has been questioned by Lashley (1930) and others, and at the spinal level the structure of the cells concerned seems in itself sufficient to deny the value of this explanation of inhibition. The terminal branching of the axon makes it difficult to see how the activity of one branch could influence the transmission of impulses from another branch. It is suggested, that is, that the axon A, with two terminal branches A’ and A” (Figure 5B) activating B and C, will with a greater activity of B tend to activate B and not to activate C. Here the only way such an event could occur is by the effects of the disturbance in B, which could presumably affect ionic concentrations in its immediate neighborhood, passing back along A’ and making A’ more readily excitable than A.” There is no reason for supposing that this could occur. It seems slightly more plausible to postulate an internuncial neuron D, whose cell body is in contact with that of B, so that A’ might tend to activate D alone, this leading to the greater activity of C. Histologically this seems quite possible, in view of the wealth of connections in the ventral columns. But the difficulty is thereby only removed to a new locus. The “boutons terminaux” of A’, in contact with B and D, are themselves distinct as A’ and A” are distinct, and the problem is unsolved. *Yet, here Hebb in 1934 seems to be hypothesizing the closed loops of Cajal and Lorente de Nó, which he did not “discover” until 1944!

**Figure 4:**
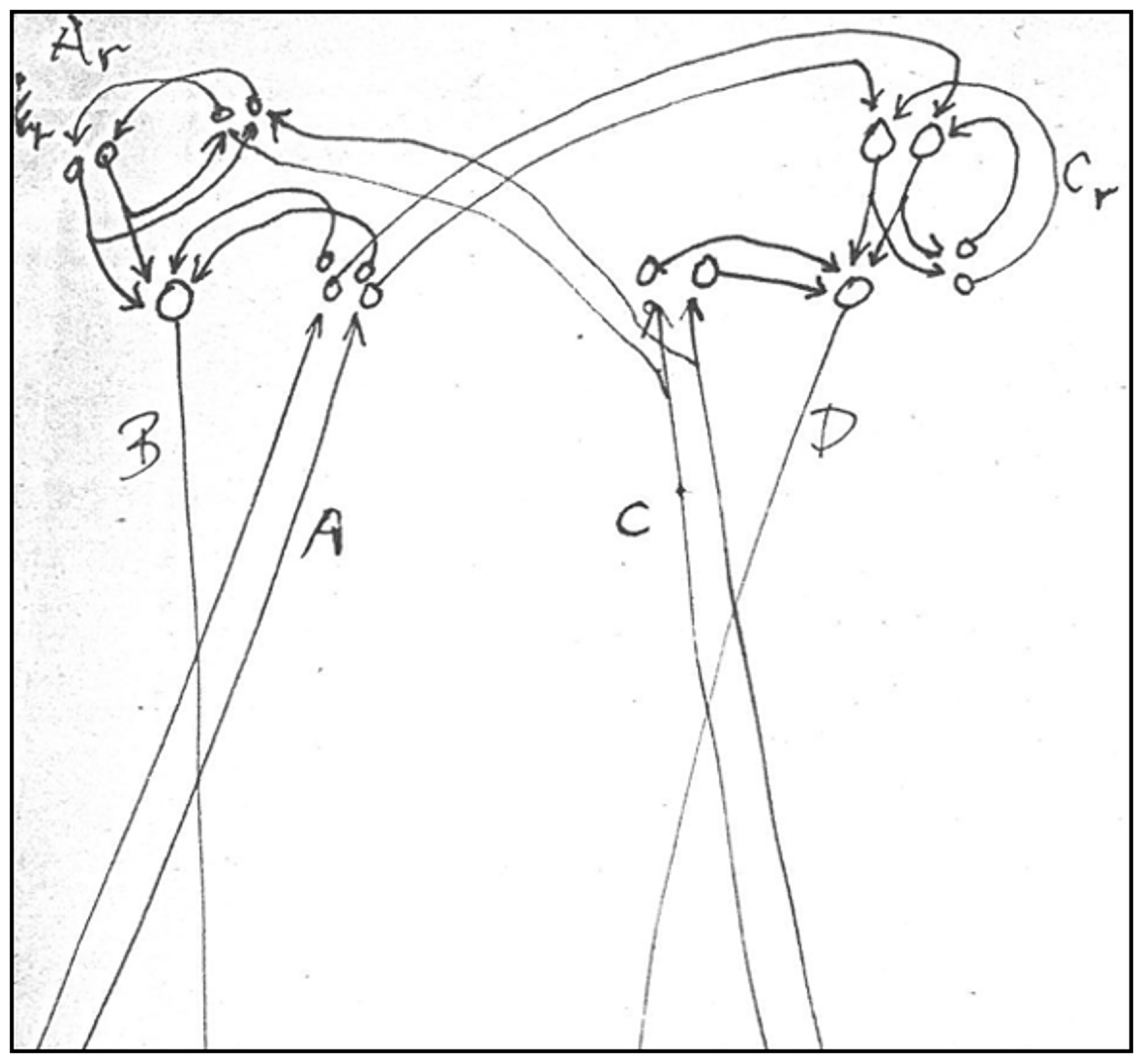
Hebb’s (1934) depiction of a possible mechanism of reflex inhibition. Hebb’s (1934) explanation for this figure was that: “on the assumption that afferents A fire B only when a supporting or reinforcing activity is going on in the reticular substance (A_r_ = A reinforcing)—and that the afferents C fire A_r_ completely. It is necessary to assume also that this sudden complete firing of A_r_ does not add to the normal level of effectiveness of A on B, since the onset of inhibitory action is not heralded by a preliminary increased activation. It is also necessary to assume that the internuncial connections of A to A_r_ are essential to the reinstatement of activity in Ar, and hence that A_r_ does become active again when A is reactivated, but not until then. Thus, the cumulative loss of tonus postulated by my term paper of ‘34 is still essential as the matter stands at present.

Hebb’s earliest ideas on the neurobiological basis of behavior were developed in a series of papers between 1932 and 1934: his 1932 MA thesis which included his theory of conditioned inhibition; his 1934 paper on the interpretation of experimental data on neural action; and his 1933–34 paper on scientific method in psychology. He also used data from his 1936 PhD thesis and his research on field orientation of rats in developing his concepts of cell assemblies and phase sequences when he returned his attention to his theory in 1944. These early writings show Hebb’s thinking (see Figures 1–4) before he encountered the work of Cajal and Lorente de Nó.

As mentioned above, Hebb’s first thoughts about the neural basis of behavior were in his MA thesis at McGill University (Brown and Milner, 2003) and I expand on this thesis here to correct the impression of Cooper (2005, p. 862) that the ideas in it were unrelated to the development of Hebb (1949a) cell assembly theory. In the Introduction to this thesis, Hebb (1932, page 2) stated that: “The purpose of this paper is to present a theory of the functioning of the synapse based on the experimental work of Sherrington and Pavlov, on reflexes and inhibition.” It is noteworthy that this thesis had a focus on inhibition, whereas inhibitory processes were not considered in Hebb’s (1949a) final theory and had to wait for Milner’s (1957) revisions (Brown, 2020). It remains a mystery how, where and when inhibitory processes vanished from Hebb’s theory, but some suggestions are made below. About his thesis, Hebb (1932, p. 6) said that: “It offers a simplicity of theory which is capable of explaining the experimental facts at present available, bringing conditioned and unconditioned reflexes and inhibition under one law, and yet without the inherent improbabilities of the refractory-phase theory,” and that there was “no incompatibility between the theory of this paper and that of Sherrington (1925) and Liddell and Sherrington (1925).

The first chapter, entitled “Functioning of synapses in the conditioned reflex” included Hebb’s first attempt to describe what has become known as the “Hebb synapse.” Hebb (1932, pp. 8–11) has a lengthy explanation of this idea which is summarized here in the caption to Figure 1. Hebb (1932, p. 12) went on to say that: “The discharge of one neuron into another is increased by the discharge of the second neuron” and that: “It is this generalization which makes conditioned and unconditioned reflexes, as well as reciprocal and “internal” (conditioned) inhibition appear as different manifestations of one process.” At the end of chapter I he said that: “The conclusions of this section may be stated as follows: An excited neuron tends to decrease its discharge to inactive neurons, and increase this discharge to any active neuron, and therefore to form a route to it, whether there are intervening neurons between the two or not. With repetition this tendency is prepotent in the formation of neural routes” (underlining as done by Hebb, 1932, p. 13).

**Figure 5:**
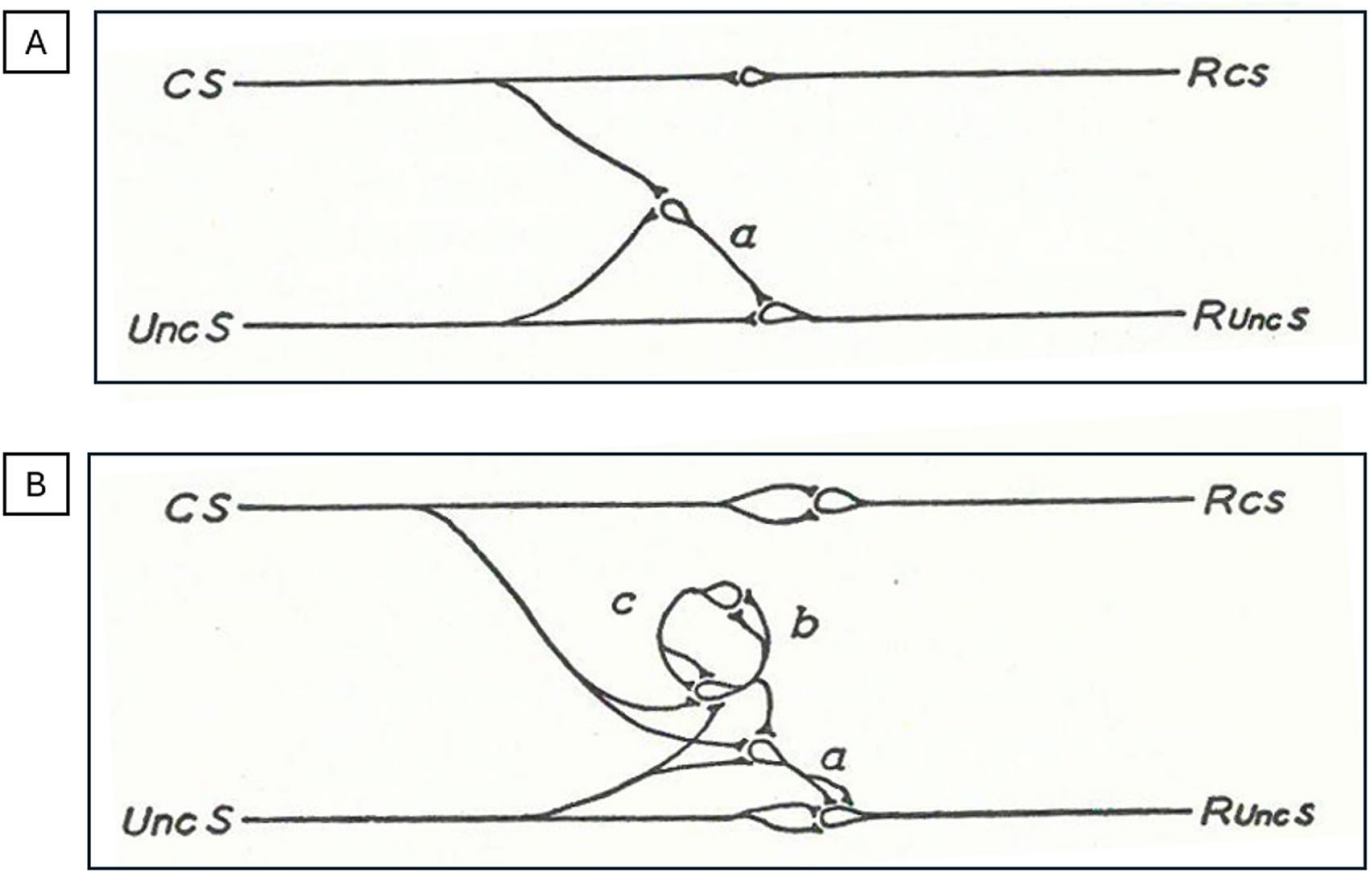
These two figures from Hilgard and Marquis (1940, Figures 38, 39, page 329) are those that showed Hebb the difference between a direct reflex route (Figure 5A) and recurrent loop (Figure 5B). **(A)** Schematic Arrangement of Neurons to Account for Conditioning by Alteration of Synaptic threshold. The caption from Hilgard and Marquis (1940, page 329, Figure 38) is as follows: “Impulses in neuron *UncS*, set up by the unconditioned stimulus, evoke a response in the neuron *R_unc_s*, which participates in the unconditioned response. Impulses in *CS* excite neuron *R_cs_* (the original response to the conditioned stimulus) but are not effective at first in exciting *a*. If *CS* and *UncS* (and therefore *a*) are simultaneously activated, there occurs an increase in the excitability of *a* such that *CS* alone is eventually able to excite it. This diagram differs from conventional ones only by the addition of the fiber *a*. If the collateral of *CS* ended directly on *R_unc_s*, the threshold change in the latter neuron would necessarily be accompanied by an increased excitability of the reflex response to the unconditioned stimulus. The present diagram permits the threshold of the conditioned response to vary independently of the original responses.” **(B)** Schematic Arrangement of Neurons to Account for Conditioning By Closed Chains. Hilgard and Marquis (1940, p. 329) state that “Recent observations indicate that the path of neural impulses from sensory to motor nerves is not single “straight-through” pathway (Lorente de Nó, 1938c) and this figure depicts the recurrent loops of Lorente de Nó. The caption from Hilgard and Marquis (1940, page 331, Figure 39) is as follows: “The assumption is made that activation of a nerve cell occurs only when the cell receives excitation from two axon endings simultaneously. The symbols are the same as in Figure 5A. Impulses in *CS* are ineffective with respect to neurons *a* and *b*. If impulses in *UncS* and *CS* arrive simultaneously, however, they summate to excite *b*. This sets the closed chain *b-c* in continuous activity, and impulses in *CS* now summate with collaterals from fibers in the chain to activate *a* and *R_uncs_*. Simultaneous excitation of *b* will occur by chance if *CS* and *UncS* are stimulated together repeatedly. The chances of simultaneity are increased if the frequency of impulses in *CS* and *UncS* is greater; i.e., if the intensity of the stimuli is greater. While the speed of conditioning in any single neuron unit is thus largely a matter of chance according to this scheme, the sum of the changes in many such units would result in a gradual increase in the number of *R_uncs_* fibers activated. This scheme is not elaborated here to account for extinction, or other phenomena of conditioning. Inhibitory effects might be introduced by the addition of specific inhibitory collaterals, or by consideration of temporal relations resulting in refractory period decrement.”

In the second chapter, Hebb (1932) discussed the development of the unconditioned reflex as an interaction between hereditary neurodevelopmental factors and early conditioning. He suggested that “Instead of inheriting either conditioned or unconditioned reflex, there is inherited a capacity for forming them.” (p. 21). In Chapter 3 on “Unconditioned inhibition,” Hebb (1932) gave a critique of the refractory phase theory of inhibition based on the papers of Forbes (1929) and Dodge (1931), and supported Sherrington’s (1925) concept of reciprocal inhibition, which he depicted in his Figure 5 (Figure 1B). To understand the state of theories of inhibition at the time that Hebb was writing, one should refer to Dodge (1926a) on the problem of inhibition and Dodge (1926b) on the refractory phase hypothesis of inhibition.

In the final chapter on “Conditioned inhibition: hypnosis, sleep, and the waking state,” Hebb (1932) examined the concept of conditioned inhibition in the cortex as was used by Pavlov (1928, chapters 30–32) to examine hypnosis and sleep. Hebb (p. 44) then applied Sherrington’s idea of reciprocal inhibition to Pavlov’s concept of “internal inhibition,” suggesting “an explanation of the general nature of inhibition” by putting “inhibition under the head of the alternation of neural routes” as shown in Figure 1B. Hebb was trying to explain inhibition as the opposite of facilitation and extended this idea “applying this generalization to the unconditioned reflex and reciprocal inhibition” (p. 13). Hebb (1932, p. 43) also considered the difference between temporary and permanent facilitation of a neural route, saying that: “permanent facilitation is regarded as a lowering of synaptic resistance which remains after impulses have crossed the synapse. The temporary facilitation remains only during (or shortly after?) the activity of the neurons which caused it; ‘Recency’, that is, is a factor distinct from ‘intensity and frequency’ “(p. 43). Here we have the problem that beguiled Hebb for some time: how do neurons remain active after the termination of a stimulus? Or, how does the synaptic activity of a neuron go from a short-term to a long-term change? This is the problem for which he later found the solution in the closed loops of Cajal and Lorente de Nó. Thus, in this early MA thesis, Hebb considered many of the issues that he was later to develop as he wrote his notes for *The Organization of Behavior*.

Much of the final chapter of Hebb’s (1932) MA thesis focused on using his concepts of the neural basis of inhibition (see Figures 1A,B) to explain Pavlov’s four types of internal inhibition. In his Figure 8 (Figure 2A), Hebb (1932) diagrammed his concept of reciprocal inhibition and suggested that a similar neural mechanism could underlie inhibition of delay (Pavlov’s second type of internal inhibition), which might underlie hypnotic states. Hebb went on to argue that the same mechanism could operate in the third and fourth types of internal inhibition described by Pavlov (1928, page 238): differential inhibition and conditioned inhibition. Hebb’s Figure 9 (see Figure 2B) shows how his theory would explain these two types of internal inhibition. As Pavlov (1928) had related both sleep and hypnosis to inhibitory mechanisms, Hebb (1932, p. 52) then applied his theory of inhibition to sleep and hypnosis, saying that “Hypnotism and sleep are so intimately related to internal inhibition that Pavlov regards them as being merely forms of a widespread inhibition.” And that is why Hebb (1932) extended his model of inhibition to sleep and hypnotic states.

While a graduate student at the University of Chicago in November 1934, Hebb reworked the ideas from his 1932 MA thesis into an 18-page essay for his class in Anatomy 316, which was taught by C. J. Herrick. In this paper, Hebb (1934, page 1) expanded on the ideas about inhibition outlined in his MA thesis (see Figure 3). Hebb argued that Pavlov and Sherrington conceived of a reflex as having a “fixed route,” “the assumption of relatively unchanging paths along which an excitation peripherally aroused is conducted” (p. 2), and noted that “The evidence for this inhibitory action again requires the notion of specific routes in order to be conclusive” (Hebb, 1934, p. 5). Hebb then examined different theories of inhibition, including the drainage hypothesis of McDougall (1926), the concept of reciprocal inhibition (Sherrington, 1925, 1929), the refractory phase hypothesis; synaptic fatigue, and Pavlov’s (1927, p. 246) concept of internal inhibition. Referring back to the ideas on conditioned inhibition in his 1932 thesis, Hebb (1934, page 9) gave some support to McDougall’s (1926) drainage theory, but concluded that “The present information about cortical function shows it apparently so complex and so little understood as to make such assumptions in cortical inhibition far from safe, and of very doubtful value.” He then went on to discuss the evidence concerning spinal inhibition, relying on the work of Sherrington (1925) and Liddell and Sherrington (1925) and produced a new drawing to describe his concept of reciprocal inhibition (See Figure 4). This 1934 paper was never finished, but these ideas stayed with him (as did his unpublished papers) and he used them in 1944, when he began to think about a schema for a neurophysiological theory of behavior. It appears that this paper was the last time that Hebb tried to make sense of inhibition.

While a graduate student at McGill and later in Chicago, Hebb (1933–34) wrote five chapters (69 pages) of a manuscript entitled “Scientific Method in Psychology: A Theory of Epistemology Based on Objective Psychology.” In the introduction to this manuscript, he wrote that his aim was to examine the question: “Is epistemological theory affected by the physiology of Pavlov, the “behaviorism” of Thorndike or Watson, or by the work done by Köhler and Lashley?” In this manuscript, Hebb (p. 24) stated that although Psychology was often considered to be the study of the mind by the process of introspection, “Psychology is not the study of mind, any more than Physics is the study of relativity, but of man’s activity, or behavior.” He argued that the introspective study of mind in psychology must give way to a more objective study of behavior based on the knowledge of the brain and the principles of physiology. Hebb continued to work on this manuscript at the University of Chicago and it contains comments from a philosophy student at Chicago, Warner A. Wick (who completed his PhD thesis on “Metaphysics and the new logic” in 1941 and became a professor of philosophy at the University of Chicago), but nothing seems to have become of this paper until 1980 when he revived some of the ideas (tempered by almost 50 years of intervening experience in psychology) for his final book “*Essay on Mind*” (Hebb, 1980b).

In this manuscript Hebb (page 39) was struggling to derive a physiological theory of mind and behavior. He said that: “In order to understand human activities in terms of physiological theory it is not sufficient to substitute the words ‘conditioned reflex’ for ‘association’, and ‘brain’ for ‘mind’, thinking of the brain as a distinct organ receiving impulses from different routes, each route indicating some distinct sensory perception. The modernizing of older forms of thought has sometimes been rather ingenious. As soon as the neuron-synapse theory began to make it possible to think of a completely neural determination of human activity, it was assumed frequently that cortical or at any rate cerebral processes alone correlated with the process called conscious. There may be no consciousness without the cortex; but there is no reason to conclude that in the presence of the cortex the functioning of sensory and sub-cortical neurons have nothing to do with ‘conscious’ processes.” Hebb does not seem to have finished this 1933–34 paper, but in it he was clearly struggling to develop a theory of the neurophysiological organization of behavior.

Hebb began his PhD research on the field orientation of rats following cortical lesions at the University of Chicago (Hebb, 1938b, 1938c), but when he moved to Harvard, he completed his PhD on a different topic, the effects of rearing rats in the dark on visual perception, which was published in three papers on “The innate organization of visual activity” (Hebb, 1937a, 1937b, 1938a). He then spent the year 1936–37 as a teaching assistant at Harvard, got married, had a 2 year post-doctoral fellowship with Wilder Penfield in Montreal, had two children, got a job teaching Psychology at Queen’s University in Kingston, Ontario (1939–42) and then moved to Florida (1942–47) as described in his autobiographical chapters (Hebb, 1959a, 1980a). Hebb never gave up on his theory of the neural basis of behavior, but this theory was put on the back burner while he did his bread-and-butter work in Montreal, Kingston and Orange Park. Between 1939 and 1945 Hebb wrote papers on the results of neuropsychological tests on the patients of Wider Penfield and developed his ideas on the nature of intelligence. He also developed new tests of adult intelligence (Hebb and Morton, 1943), wrote about the effects of early and late brain injury on intelligence (Hebb, 1942) and published his review of frontal lobe function (Hebb, 1945a). From 1942 to 1947 he published the results of his studies on temperament and emotional expression in chimpanzees which he conducted in Florida (Hebb, 1945b, 1946a, 1946b, 1947a, 1949b) and began work on his book, to be called *The Organization of Behavior*.

## 5 Hebb’s comments on how he discovered Lorente de Nó’s work on recurrent loops

While writing his papers on the effects of early and late brain injury on intelligence (Hebb, 1942) and the functions of the frontal lobes (Hebb, 1945a), Hebb began to think about the neural basis of intelligence. In his Hughlings-Jackson lecture, Hebb (1958, p. 266) stated that “About this time, also, Lorente de Nó’s conceptions of synaptic function and of reverberatory circuits were becoming known. Hilgard and Marquis (1940) had drawn attention to some of the possibilities of the reverberatory circuit for psychological theory and it seemed that Lorente de Nó (1939) idea of the need of summation at the synapse provided a clue to the selective operation of ‘set’ or ‘attention’. On this foundation, then, my attempted solution for the problem of higher mental processes (Hebb, 1949a) was built.” “The long and short of the theory is that an elementary idea consists of activity in a complex closed loop called a “cell-assembly,” developed as the result of repeated stimulation during childhood; that one or more of these, simultaneously active, can excite another, in a series called a “phase sequence”—that is, a train of thought—and that Lorente de Nó’s need of summation for synaptic transmission, in the operation of these cell assemblies, accounts for the selectivity and directedness of the train of thought.”

Hebb (1980a, page 292) said that “more brain tissue or more raw intellectual power is necessary to develop a concept than to retain it. What then is a concept in terms of neural mechanisms? And at this point my thinking stalled, partly because, like everyone else, I was still thinking of the brain as a through-transmission device and partly because of difficulty in reconciling the facts of learning (which must be localized in specific synapses) and the facts of perception (which, it seemed, are not localized). I had given up thinking about the problem for 2 years or so, when reading Hilgard and Marquis (1940) drew my attention to Rafael Lorente de Nó’s work and led me to write *The Organization of Behavior*, which contained a theory quite different from any of my earlier ideas [from 1932–34].” Hebb (1980a, page 295) described how he used the ideas of Lorente de Nó to develop his theory: “It was in February 1944 that I came back to the problem of thought and the brain, when I found out that Rafael Lorente de Nó had recently shown: (1) that reentrant or closed circuits were to be found throughout the brain, which thus was no longer to be seen as a through-transmission, sensorimotor mechanism, but as one capable of a purely internal activity also, as a possible basis of thought; and (2) that one neuron by itself may not be able to excite a second neuron at the synapse, but can do so if supported by simultaneous action from another neuron.”

In their textbook, *Conditioning and Learning*, Hilgard and Marquis (1940, pages 326–334) included a section on “The Nature of Synaptic Modification” in which they cited three papers by Lorente de Nó (1938a, 1938b, 1938c) and presented two figures (see Figures 5A,B) showing how synaptic change could underlie learning. It is worth summarizing this section of their book as it was instrumental in altering Hebb’s thinking about the neural basis of behavior. Their aim was to examine “the nature of the actual neural modifications” underlying conditioning” (p. 326) and they proposed that in conditioning there is (1) “a relatively permanent decrease in the threshold for evoking the response by the conditioned stimulus,” and (2) “this change in threshold must be localized at synaptic junctions between neurons” (p. 326–327). They discussed two plausible theories for such events: the development of new synapses, and changes in the physio-chemical properties of the nerve cell. In their Figure 38 (see Figure 5A), they described how the simultaneous excitation of fiber *a* by two axons somehow produces a long-lasting decrease in the threshold” at the synapse. They pointed out that “Lorente de Nó (1938b), “has demonstrated that spatial summation of at least two simultaneous impulses is necessary to excite a neuron under any conditions” (p. 329). Secondly, they summarized the results of Lorente de Nó (1938c) in which he described closed chains of neurons “in which a collateral excites a circle composed of several neurons” in which “the chain of neurons may maintain its activity indefinitely in the sense of peripheral afferent impulses” (p. 330). In their Figure 39 (Figure 5B), they showed how such a closed circuit might be a mechanism of learning. “Closed chains, set into activity by the training procedure and continuing in the absence of any external excitation, would summate with otherwise inadequate afferent impulses to produce a conditioned response.”

With respect to theories of inhibition, Hilgard and Marquis (1940, p. 334) stated that “Current neural theories [of inhibition] are perhaps in greater flux than ever before, and it is beyond the province of this discussion to attempt to evaluate them.” This, and the comments of Lorente de Nó (see below), may have been the impetus for Hebb to give up on his attempts to develop a neural theory of inhibition.

Hebb (1949a, page 10) said that: “The nature of synaptic transmission in the central nervous system is also of fundamental importance for the theory of behavior. There are two radical modifications of earlier ideas: transmission is not simply linear but apparently always involves some closed or recurrent circuits; and a single impulse cannot ordinarily cross a synapse--two or more must act simultaneously, and two or more afferent fibers must therefore be active in order to excite a third to which they lead. The concepts of neural action chiefly developed by Lorente de Nó (1938a, 1938b, 1939, 1943) are well enough known by now to need no elaborate review. It is necessary, however, to point out that they have revolutionary implications for psychological theory.”

**Figure 6:**
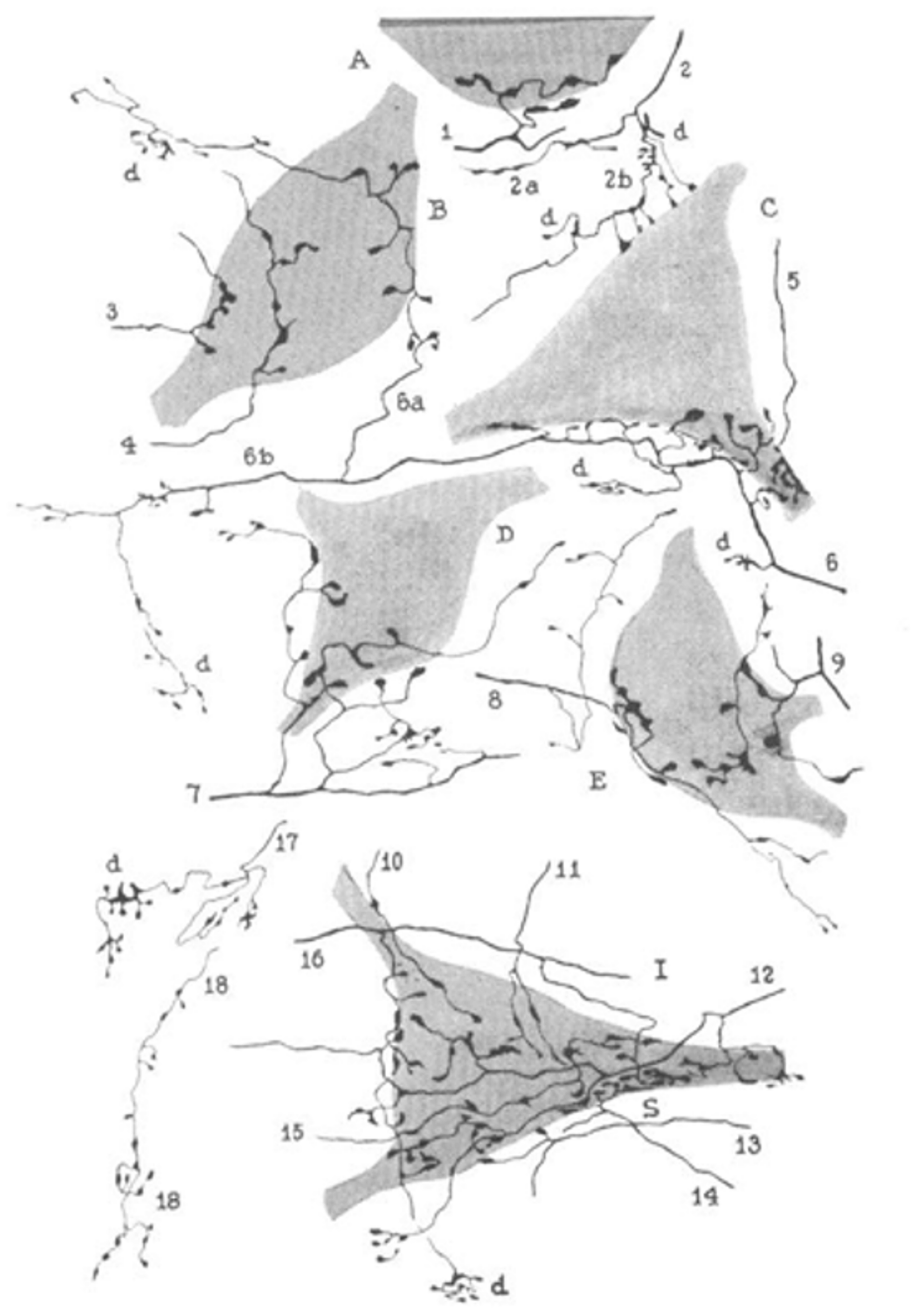
Hebb (1949a, page 64) used this figure from Lorente de Nó (1938b) to depict the relationships between synaptic knobs and the cell body, but he did not give much of an explanation of the figure, which was given a lengthy description by Lorente de Nó (1938b, pages 199–202). The original drawing of Lorente de Nó (1938b, page 200, Figure 3) showed synapses on motoneurons (A to E) and on a large internunceal (I) of the spinal cord of a 15–16 day old cat, using the silver chromate method of Golgi. In his description of this figure, Lorente de Nó pointed out that the motoneurons received synaptic knobs from many different cells. For example, Cell C received synaptic knobs from fibers 2, 5, and 6. It is unfortunate that Hebb did not go into more detail about this figure, about which he said (p. 65), “The details of these histological speculations are not important except to show that the mechanism of learning discussed in this chapter is not wholly out of touch with what is known about the neural cell.”

Hebb (1949a, p. 61) explained how the recurrent circuits of Lorente de Nó could be involved in memory: “Hilgard and Marquis (1940) have shown how a reverberatory, transient trace mechanism might be proposed on the basis of Lorente de Nó’s conclusions, that a cell is fired only by the simultaneous activity of two or more afferent fibers, and that internuncial fibers are arranged in closed (potentially self-exciting) circuits. Their diagram (See Figure 5B) is arranged to show how a reverberatory circuit might establish a sensori-motor connection between receptor cells and the effectors which carry out a conditioned response.” In Hebb’s (1949a, page 64) book he explained how repeated stimulation of a neural circuit could facilitate the growth of the “synaptic knobs” described by Lorente de Nó. Hebb (1949b, pages 71–76) then explained how the concept of recurrent circuits could be applied to his cell assembly theory (see Figure 6).

**Figure 7:**
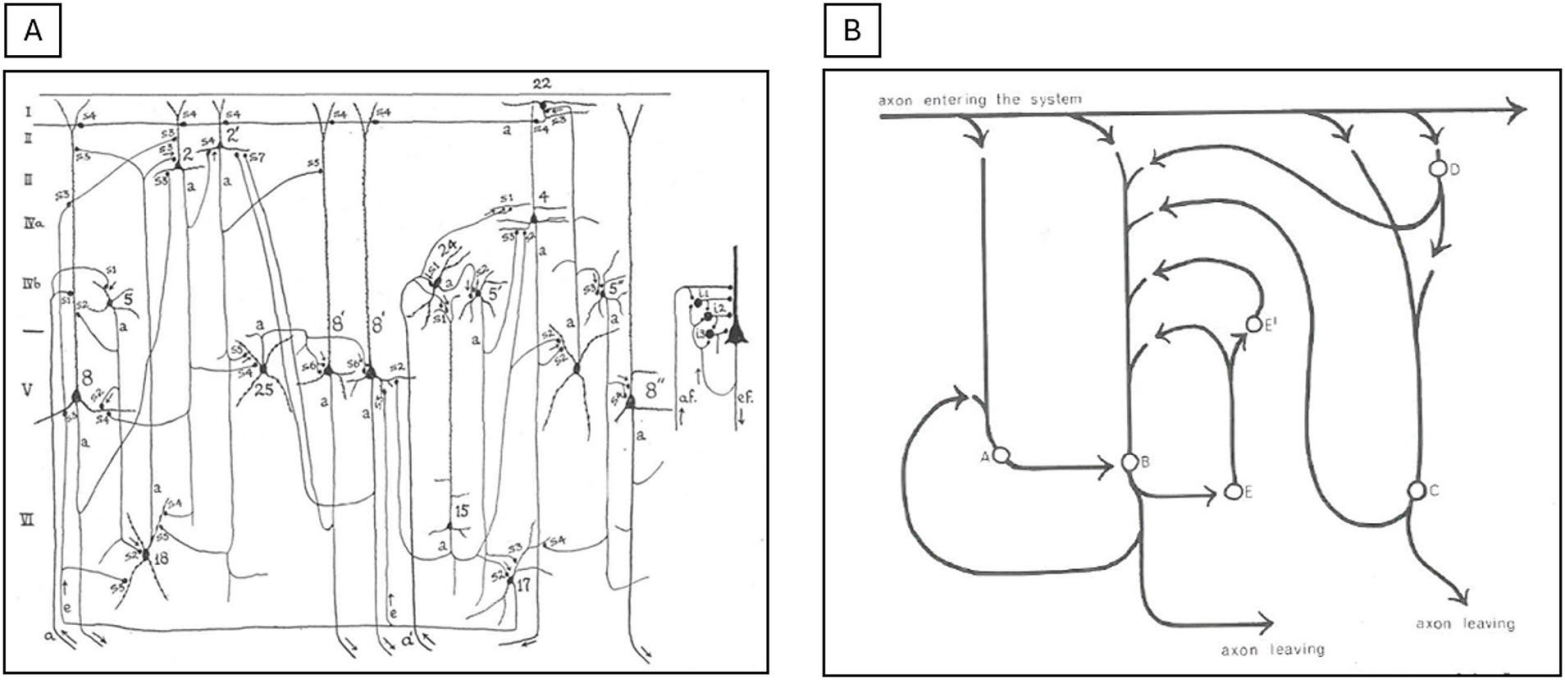
**(A)** Figure 16 in Hebb’s (1958) *Textbook of Psychology* (first edition, page 56) showing a diagram of a closed path in the human cortex. The small diagram at the right is a simplification of the larger one. Arrows show the direction of transmission in different cells. The original drawing was in Lorente de Nó (1938d, Figure 66 page 313; also in Fulton (1943), page 307, Figure 74). It is unfortunate that Hebb did not add more details about this figure, which Lorente de Nó (1938d, page 313) described as follows: Diagram of some of the intracortical chains of neurons from the parietal cortex of the adult mouse using Nissl stain. Roman numerals I -VI represent the layers of the cortex. The numbers (8, 8′, 8″) depict large pyramidal cells of layer V. The axons of the cortical cells are marked with *a*. Note that only a few dendrites and axonal branches have been included in the diagram. The synaptic junctions are indicated with the letter *s (s1, s2*, etc.) and with a thickening of the axon. It is assumed that the synapses marked with an arrow are passed by the impulses. The small diagram at the right is a simplification of the diagram at the left. The afferent fiber *af*, activates the large pyramid which is the origin of an efferent fiber *ef*, and also a system of cortical internuncial cells *(i1, i2, i3*); the recurrent collateral of *ef* delivers impulses again to the internuncial system. This diagram summarizes the plan upon which the central nervous system is built. **(B)** In the second edition of his *Textbook of Psychology*, Hebb (1966, page 77, Figure 35) gave a diagram of relations between neurons actually observed by Lorente de Nó (based on the figure in Lashley, 1949). In this figure, the entering axon excites the dendrites of four neurons, A, B, C, and D. Of these, B and C send impulses out of the system to excite other systems, but impulses from A and D are delivered only within the system itself. A-B, B-E, and B-E–E’ form closed circuits which can hold excitations and cause B to continue delivering impulses outside the system. These two figures are also published in the fourth edition of the Textbook (Hebb and Donderi, 1987, pages 78–79, Figure 4.7 b and c).

In his first autobiographical paper on his neuropsychological theory, Hebb (1959a) did not mention Lorente de Nó, but stated that the cell assembly “is a closed system in which activity can ‘reverberate’ and thus continue after the sensory event which started it has ceased.” (p. 628). In the first edition of his *Textbook of Psychology*, Hebb (1958, p. 55–58, Figure 16) discussed reverberatory circuits and included a diagram from Lorente de Nó (1938d, Figure 66; also Fulton (1943), Figure 74; see Figure 7A), but this figure was not explained very well. The second edition of the *Textbook* (Hebb, 1966, pages 72–80) discussed the reverberatory circuits of Lorente de Nó and, in addition to the figure (now Figure 34) from Fulton (1943), Hebb added a schematic diagram of a reverberatory circuit as Figure 35 (Figure 7B). In a new section at the end of this edition, Hebb (1966, page 316) discussed the importance of the reverberatory loops of Cajal and Lorente de Nó but did not provide any references. He said that: “When Thorndike and Watson and E. B. Holt were establishing S-R theory, the nervous system was thought to be a set of paths running from receptor to effector, some longer and less direct (i.e., through the cortex, in learning), but all one way streets; no back connections, no feedback within the system, no loop circuits in which an excitation could maintain itself without sensory stimulation. The self-exciting paths of Figures 34 and 35 (Figures 7A,B) had been described by the great Spanish anatomist S. Ramon y Cajal but physiology had paid no attention to them. For physiologists as much as for psychologists, all neural transmissions had to be straight through, from one sense organ to muscle or gland. The alternative, that some other activity might go on inside the brain, seemed to be denied by hard facts. Thus, theoretical development was at an impasse until R. Lorente de Nó again demonstrated Ramon y Cajal’s closed circuits and this time got the attention of physiologists and psychologists, about 1940.”

Hebb (1966, page 321–322) ended the second edition of his textbook by noting that: “E. R. Hilgard and D. G. Marquis, *Conditioning and Learning* (Appleton-Century- Crofts), were the first to note some of the new possibilities opened up by the anatomical and physiological ideas of Lorente de Nó (p. 331 in their 1940 edition; such foreign matter has disappeared from the revision by G. A. Kimble (1961).” The fourth edition of the textbook (Hebb and Donderi, 1987, p. 81) expanded the references to Lorente de Nó, stating that “Much of the CNS is filled with neural paths that lead back into themselves. Figure 4.7b (from Fulton, 1943, shown as Figure 7A) shows this in schematic form. It is a drawing by R. Lorente de Nó, the distinguished neuroanatomist and physiologist to whom we owe most of our original knowledge of these matters.” Although *The Organization of Behavior* referenced four papers by Lorente de Nó, none of the four editions of Hebb’s textbook included references to any of Lorente de Nó’s papers! The figures related to Lorente de Nó came from Fulton (1943) or Beach et al (1960). Lorente de Nó was cited in the Author Index of the *Textbook*, but no proper reference citations were given to the important and inspiring papers of Lorente de Nó. In his *Essay on Mind*, Hebb (1980b, page 87) again explained the importance of Lorente de Nó’s ideas in the development of his neurophysiological theory by saying that “it had become easy to see how the central representative processes could become associated, if the two stimuli excited activity in reverberatory closed pathways and not mere straight-through transmission,” but, once again, he did not cite any of Lorente de Nó’s original papers.

In addition to Hilgard and Marquis (1940) book *Conditioning and Learning*, there were at least three other sources for Hebb’s discovery of the importance of Lorente de Nó’s work for his theory: Morgan, Fulton, and Lashley.

Hebb (1949b, page vii) mentioned and cited Morgan’s (1943) textbook *Physiological Psychology* and said that “Of these [reference sources] Morgan is the most directly relevant,” particularly for Hebb’s conception of the central motive state and the “problems of motivation: pain and hunger” (Chapter 8 of *The Organization of Behavior)*. The second less mentioned source was Fulton’s (1943) textbook, *Physiology of the nervous system.* Hebb (1949a) referenced Fulton (1943, second edition), which has considerable information about recurrent circuits and other information from Lorente de Nó, which Hebb used. The third edition revised (Fulton, 1949) has a discussion of Lorente de Nó’s work on temporal and spatial summation of excitatory stimuli at a synapse. For temporal summation, Fulton (1949, p. 63) said that: “When two stimuli, each of them too weak to evoke a response, are applied to the same nerve trunk, the second inadequate stimulus may evoke a response if applied at a brief interval after the first because of the enduring character of the local excitatory process.” For spatial summation, Fulton (1949, p. 64) said: “Spatial summation is of primary importance in the physiology of the nervous system. If successive stimuli are applied to two different nerves which play upon the same reflex center, summed effects can readily be demonstrated.” Fulton (1949) contains an extensive discussion of the work of Lorente de Nó in Chapter 15, “Cerebral cortex: Architecture, intracortical connections, motor projections”, where Figure 74 (page 307) and the discussion of recurrent circuits was most useful for Hebb’s theory, and which, in the third edition revised, had the first three sections (pages 274–301) prepared by Rafael Lorente de Nó himself.

It is clear that Lashley had considerable influence on Hebb’s ‘discovery’ of the work of Lorente de Nó. Both Bruce (1998) and Nadel and Maurer (2020) allude to Lashley’s influence on Hebb’s theory, thus it is worth noting Lashley’s references to Lorente de Nó that Hebb must have read between 1937 and 1949. In a paper in which he argued against the concept of localization of function in the brain and that Hebb referred to in his “Spring ‘45″ notes (see below), Lashley (1937, page 380), said that “the recent studies of Lorente de Nó (1934b) showed a mechanism for recurrent excitation in the *cornu ammonis* which seems especially adapted for summation [of nerve impulses].” Lashley (1938, page 466) in a paper referred to by Hebb (1946a), said that “The recent demonstration of recurrent nervous circuits, perhaps capable of indefinite reverberation, by the anatomic and physiologic studies of Lorente de Nó, relieves us from the necessity of finding a peripheral mechanism to account for the maintenance of activity or for the dynamic tensions which are implied by the phenomena of motivation.” Lashley (1941a, page 335) in a paper on migraine, said that: “The drift of the scotomatous area is what might be expected from the spread of excitation across a succession of reverberatory circuits, as described by Lorente de Nó (1938c), with the activity of the circuits extinguished at the inner margin of the blind area at the same rate as it is propagated to new circuits at the advancing margin.”

In a discussion of neurology and psychology, Lashley (1941b, page 463) stated that “Recent anatomic and physiologic studies however give a quite different conception of the integrative processes within the central gray matter. An advance of prime importance was the report by Lorente de Nó (1934a) of the intricacy of connections between the cells of the hippocampal cortex. Instead of one-way transmission over limited paths, his figures show the anatomic possibility of diffuse spread of excitation through nervous tissue, almost as through a continuous network. In addition, return circuits make possible a recurrent excitation which will maintain activity for various lengths of time and, of course, greatly complicate the pattern of excitation. The action of such recurrent or reverberatory circuits has been demonstrated experimentally by Lorente de Nó (1938c)". Lashley (1942) noted that “A stimulus typically activates millions of neurons, and behavior is the statistical outcome of this activity in which the contribution of any single neuron is negligible. A reasonable structural basis for this activity is found in the reverberating network system described by Lorente de Nó.”

**Figure 8:**
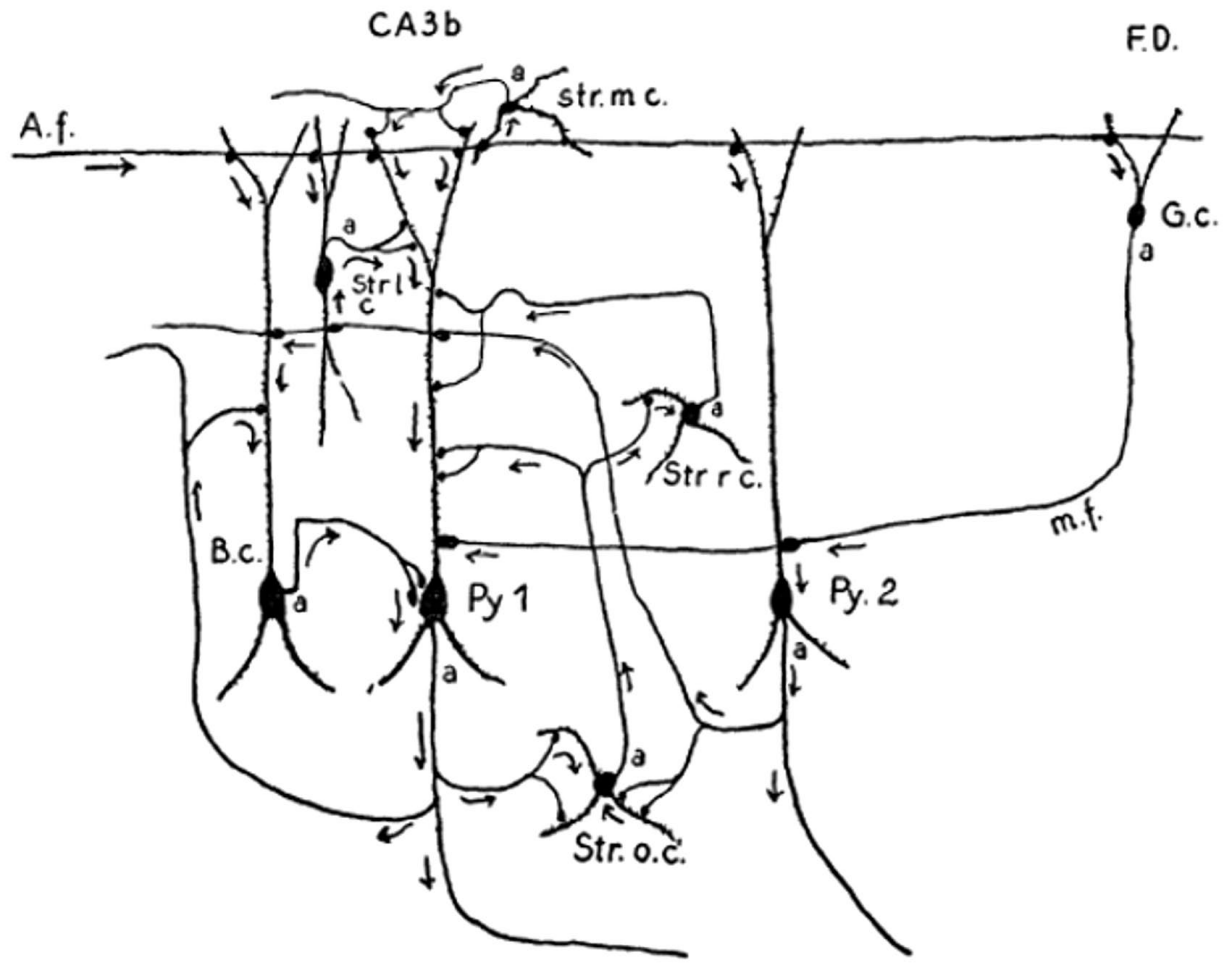
This figure from Lashley (1949; Figure 4) was taken from Lorente de Nó (1934b, Figure 35) and shows the connections between a single afferent fiber (*A.f.)* and an efferent pyramid (*Py. 1*) of the ammons horn, illustrating the complexity of recurrent connections in the cortical lattice.

Lashley had attended the same meeting as Lorente de Nó in 1946 and in his address to the American Society of Naturalists on 31 Dec 1947 (published as Lashley, 1949), he sounds very much like Hebb in describing the new concepts of the reflex: “In its functional organization, the nervous system seems to consist of schemata or basic patterns within which new stimuli are spontaneously fitted. Even in the case of learned activities the learning may, and perhaps most frequently does involve the organization of a new generalized schema rather than the formation of a limited association” (p. 35). He went on to say (p. 35–36) that “The physiology of such organizations is far from being understood, but anatomic studies suggest that a network or lattice of nerve cells forms the basis of the central integrative processes. The evidence for this comes chiefly from the work of Lorente de Nó (1934a, 1934b), which is epoch-making for the understanding of the action of the central nervous system. The cerebral cortex consists of a network of nerve cells connected together in circuits of different lengths. Lashley (1949, page 36) Figure 4 (see Figure 8) shows some of the interconnections between the cells of the cortex. An excited cell transmits impulses to adjacent cells, and the excitation may be returned from various distances, through one or a thousand links. Since the activation or inhibition of a cell is dependent upon the number and frequency of the impulses transmitted to it, it is obvious that a system so organized will have its own inherent patterns of response.” It is of interest that Hebb (1944a, 1945c) unpublished notes (see below) refer to his ‘schema’ and that Hebb (1946c) in his first draft of *The Organization of Behavior* referred to the cell assembly as a “lattice”; so did Lashley borrow these terms from Hebb, or vice versa?

Lashley (1951) gave a longer discussion of reverberating circuits in his paper on “The problem of serial order in behavior”, which he had presented at the Hixon symposium held in September 1948, at which Lorente de Nó, Köhler, McCulloch, and others also gave presentations (See Jeffries, 1951). In this paper, Lashley (1951, page 126) stated that “T. Graham Brown (1914) first showed by his studies of deafferented preparations that the rhythmic movements of respiration and progression are independent of peripheral stimulation and are maintained by a central nervous mechanism of reciprocal innervation. He suggested that this mechanism of reciprocal innervation, rather than the simple reflex, is the unit of organization of the whole nervous system. He thus foreshadowed, in a way, the conception of reverberating circuits which is coming to play so large a part in neurological theory today.” Later in the same paper Lashley (1951, page 131) said that “The cortex must be regarded as a great network of reverberatory circuits, constantly active. A new stimulus, reaching such a system does not excite an isolated reflex path but must produce widespread changes in the pattern of excitation throughout a whole system of already interacting neurons.” Nadel and Maurer (2020, page 5), found it difficult to determine whether Lashley was first to ‘discover’ the reverberating circuits of Lorente de Nó and tell Hebb about them, or whether Hebb developed the idea, which Lashley then incorporated into his own writings. However, the fact that Lashley referenced Lorente de Nó as early as 1938, and Hebb’s notes on Lorente de Nó from 1944 refer to Lashley’s papers, suggests that Lashley attended to the recurrent circuits of Lorente de Nó before Hebb.

## 6 The neuron drawings of Cajal and Lorente de Nó

It was the drawings of Cajal that influenced researchers to support the neuron theory (See López-Cantos, 2016). As pointed out by de Rijcke (2008, p. 288): “Cajal’s daily observational and experimental practices were intimately tied up with techniques of visualisation.” Likewise, Lorente de Nó produced exquisite photographs and line drawings of the cells and neural networks that he was studying, and it was on these drawings that Hebb’s (1944a, 1944b) unpublished notes were focused (see Appendix 1). Many of Cajal’s images are reproduced in Newman et al. (2017) and representative drawings of Cajal and Lorente de Nó are published in DeFelipe (2010). Cajal published his *Textura del sistema nervioso del hombre y los vertebrados (Texture of the nervous system of man and vertebrates)* in three volumes between 1899 and 1904 (Cajal, 1899-1904) and his *Histologie du système nerveux de l’homme et des vertébrés* (Translated into French by Dr. L. Azoulay) was published in two volumes between 1909 and 1911 (Cajal, 1904/1972). An English translation of this French translation, *Histology of the Nervous System of Man and Vertebrates*, was published by Swanson and Swanson in two volumes (Cajal, 1904/1995) and an English translation by Pasik and Pasik in three volumes (Cajal, 1904/2000), which also contains numerous notes on the text and a discussion of the confusing nature of Cajal, 1899-1904) book and its various translations.

**Figure 9:**
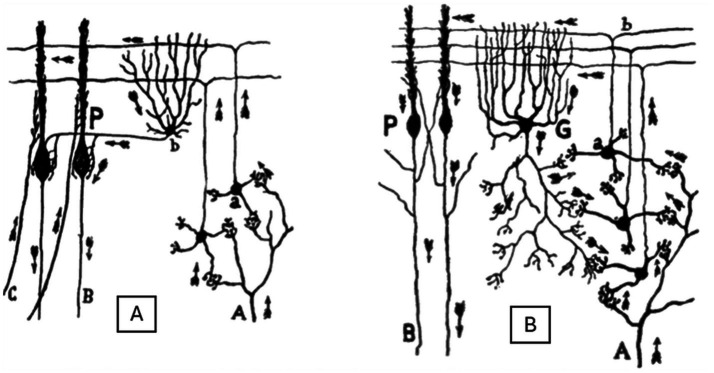
**(A,B)** This figure is from Lorente de Nó (1933, page 247; Figure 2) was originally published in Cajal (1904/1995, pages 120-121, Figures 103, 104). It shows two possible paths for the impulses carried by the mossy fibers to the cerebellar cortex; the arrows indicate the direction of transmission of the impulses. **(A)** Shows the unidirectional arc: *A*, mossy fiber; *B*, axons of Purkinje cells; *C*, climbing fibers; *a* granule cells; *b*, basket cells, and *P*, Purkinje cells. **(B)** Represents the recurrent arc through the Golgi cells (*G*). Lorente de Nó (1933, page 247) noted that Cajal had found that “certain special cells or fibers are present which establish a new kind of transmission,” which he described as follows: “A good example is offered by the cerebellar cortex (Figure 9) in which the impulses conducted by the mossy fibers are transmitted first to the granular cells and then to the basket and Purkinje cells. This transmission system (Figure 9A) corresponds to the unidirectional elementary reflex arc; but the parallel fibers also excite the Golgi cells (Figure 9B) which in their turn again excite the granular cells; these impulses go in a direction opposite to that of the former ones, and arrive again at the cell origin of the disturbance. On the other hand, the impulses set up by the granule cells arrive at the Purkinje cells twice, once through the parallel fibers (Figure 9B) and then through the basket cells (Figure 9A). Similar examples are found in the cerebral cortex, olfactory bulb, etc. However, the existence of such complicated circuits has been disregarded, undoubtedly because they seemed to be present in the highly complicated organs, such as the cerebral and cerebellar cortex; in the spinal cord, medulla oblongata and midbrain, where neither cells with short axis-cylinders nor recurrent (centrifugal) fibers had been described, only elementary [spinal reflex] arcs could be considered.”

What is important with respect to Hebb’s neuropsychological theory are Cajal’s (1904/1995) descriptions of his Figures 103 and 104 [on pages 120–123 in the Swanson and Swanson (1995) edition; Reprinted here as Figure 9]. Cajal (1904/1995) said that: “There are a large number of cells with a short axon in the cerebellar cortex. In fact, mossy fibers do not end directly on Purkinje cells; instead, they employ granule cells and their parallel fibers to relay impulses to the vast Purkinje cell dendritic tree. Furthermore, parallel fibers encounter dendrites of stellate cells in the molecular layer, as well as Golgi cells in the granular layer along their way. One may follow the course of impulse flow through these neurons in Figures 103, 104.” (p. 120). “Most currents reaching Golgi cells do so by way of their outer dendrites (Figure 103c [see Figure 9A]). The currents then descend along the short axon back to granule cells, thus tracing a recurrent arc [According to Pasik and Pasik’s note *dd* from Cajal, 1904/2000, page 467; “Cajal’s puzzlement is the reflection of the unknown process of inhibition at the time”]. It seems obvious that current transmitted by a particular granule cell does not return to that cell alone, but is instead distributed to a large number of other granule cells. Current gathered by the dendrites of basket cells spreads along the length of their transverse axon to discharge on a row of Purkinje cell bodies (Figure 104b [see Figure 9B]). “One other short pathway is formed by the small outer stellate cells in the molecular layer. These cells probably receive sensory inputs by way of parallel fibers and may transmit this information by way of a relatively irregular transverse course to the branches of Purkinje cell dendrites. This connection is obviously hypothetical because it is not based on direct observation.” (page 121). It is worth noting that the reproduction of Cajal’s Figures 103, 104 by Lorente de Nó (1933, page 247) states that it is a “diagram of Cajal showing two possible paths for the impulses carried by the mossy fibers to the cerebellar cortex.”

Cajal (1904/1995) was stumped in trying to figure out the function of “Golgi type II cells” with short axons; “Why do Golgi type II cells exist-- what is their function?” he asked (p. 121). Cajal (1904/1995, page 122) proposed the hypothesis that these cells “are simply distribution or association neurons” but then suggested that their primary function “appears to consist of *producing and storing neuronal energy*”. Since such cells are “quite abundant in the corpus striatum, cerebral cortex, cerebellum, thalamus, and so on, whereas they are very rare in the medulla and spinal cord”, Cajal postulated that they may be “concerned with memory, ideation, judgment, and so on, which, in the final analysis, occur well after the detection of environmental stimuli, or with activities like walking, jumping, defense, and so on, in which a weak stimulus can lead to a motor reflex that is disproportionately large in terms of strength and extent.” Cajal pointed out that these cells with short axons “Synapse directly onto cells with a long axon” and that currents flowing along cells with a short axon often trace a recurrent path and discharge onto the afferent sensory pathway generating them, as with Golgi cells in the cerebellum. [Pasik and Pasik in their note *gg* (Cajal, 1904/2000, page 468) say that “The compelling need to find a specific role for short axon neurons is here very evident. Only after the discovery of inhibitory processes, a more realistic function was initially hypothesized for these cells, with a probable synaptic network arrangement.”]

**Figure 10:**
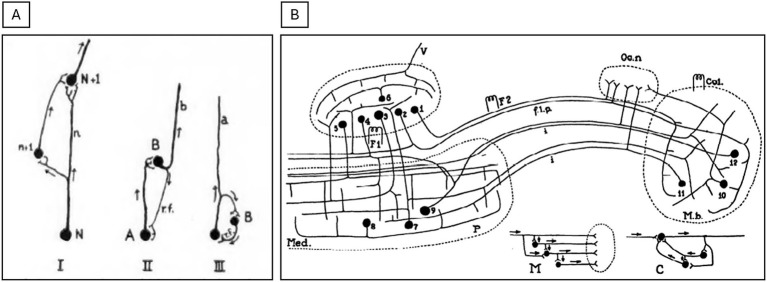
**(A)** Diagrams of the connections within the chains of neurons as depicted by Lorente de Nó (1933, page 248, Figure 3). *I* represents the law of plurality of connections: *II* and *III*, the law of reciprocity of connections. The arrows indicate the direction of the transmission of the impulses according to the law of axonal polarization (Cajal); this is also called the law of irreversibility of the synapse (Sherrington). As noted by Lorente de Nó (1933, page 248): “During the last twelve years I have been conducting studies on the structure of the nervous system with the purpose of determining whether the general plan of structure is represented by the unidirectional transmission system of Figure 9A, or rather by circuits similar to those in Figure 9B. In all systems that have been examined for their presence, cells with short axis-cylinders and recurrent fibers, either collateral or terminal, have been found, so that the following two laws are demonstrated: 1. The Law of Plurality of Connections If the cells in the spinal or cranial ganglia are called cells of the “first order” and the following ones in the transmission system cells of the second, third to. nth order, it can be said that each nucleus in the nervous system always receives fibers of at least n and n + 1 order, and often of n, n + 1 and n + 2 order. This is indicated in diagram *I* of Figure 10A. Between the nuclei N and N + 1 there exist always two kinds of pathways; one pathway n goes direct from N to N + 1; the other pathway n + 1 encloses an additional neuron; therefore, the impulses set up by the cells of N may arrive at nucleus N + 1 twice, once conducted through fibers n and a second time conducted by cells and fibers n + 1. 2. The Law of Reciprocity of Connections (**A**, *II and III)*. lf cell complex A sends fibers to cell or cell complex B, B also sends fibers to A, either direct or by means of one internuncial neuron. Cells A and B may lie in the same nucleus (**A**, *III*); in this case A is the efferent cell and B is a cell with a short axis-cylinder; in the chain A-B a third neuron may often be intercalated between A and B. In other cases, A and B lie in different nuclei (**A**, *II*); then the r.f. fibers are the so-called recurrent or centrifugal fibers. The existence of centrifugal fibers was discovered by Cajal in the retina and olfactory bulb. Later, Cajal and Probst described centrifugal fibers from the cerebral cortex to the thalamus, and recently numerous authors have confirmed and completed their descriptions. But the centrifugal fibers were considered as a peculiarity of centers of high order and therefore not as a general trait of structure of the whole nervous system. However, they may be found everywhere if appropriate methods (Golgi’s method) are used.” **(B)** As described by Lorente de Nó (1938c, page 210, Figure 2): The internuncial neurons are arranged in chains of two types: M (principle of plurality of connections) and C (principle of reciprocity of connections) [as described by Lorente de Nó (1933, page 248); and shown in **A**], which, with but few exceptions, are also found in every part of the central nervous system. This is a diagram of the pathways connecting the internuncial cells among themselves and with the ocular motoneurons. V, vestibular nerve; 1 to 6, cells in the primary vestibular nuclei; 7, 8, 9, cells in the reticular formation in the medulla (Med.) and pons (P.); 10, 11, 12, cells in the reticular nuclei in the midbrain (M.b.); Oc.n., oculomotor nuclei; f.l.p., fasciculus longitudinalis posterior and similar pathways; i, internuncial pathways; F1, F2 and Col., position of the stimulating electrodes. The diagrams below indicate the two types of chains formed by internuncial cells; M, multiple and C, closed chain. These two figures were particularly important for stimulating Hebb’s ideas on reverberating circuits, as indicated by his notes (Hebb, 1944a, see Appendix 1).

Figures 103, 104 of Cajal (1904/1995), reproduced by Lorente de Nó (1933) and Mira and Delgado (1999) were Cajal’s only reference to recurring circuits (see Figures 9A,B). Cajal was an anatomist, concerned with the structure of the nervous system, while Lorente de Nó was both an anatomist and an electrophysiologist, and thus examined both the structure and function of recurrent circuits. Mira and Delgado (1999, p. 12–14) used the work of Cajal, Lorente de Nó, Lashley and others on the importance of reverberatory loops in the functional organization of the nervous system to develop their concept of an “information loop” (feedback loop) as “the basic fundamental module in the organization of the NS [nervous system]”. They stated that: “as far as we know, Ramon y Cajal, in 1904, first gives us graphic constancy of the existence of feedback loops in the cerebellum (recurrent semicircles), through short-axoned cells (Golgi type II), that function as associative units in multiple feedback loops. These cells generate divergent and convergent pathways around the Purkinje cells, which provide the only output lines.” They show figures of the feedback loops proposed by Cajal in 1904 for the cerebellar cortex and the M chain and C chain circuits of Lorente de Nó (1938b) (see Figure 10) and relate these to “Hebbian changes in the efficiency of the synaptic transmission processes” (Mira and Delgado, 1999, p. 22).

**Figure 11:**
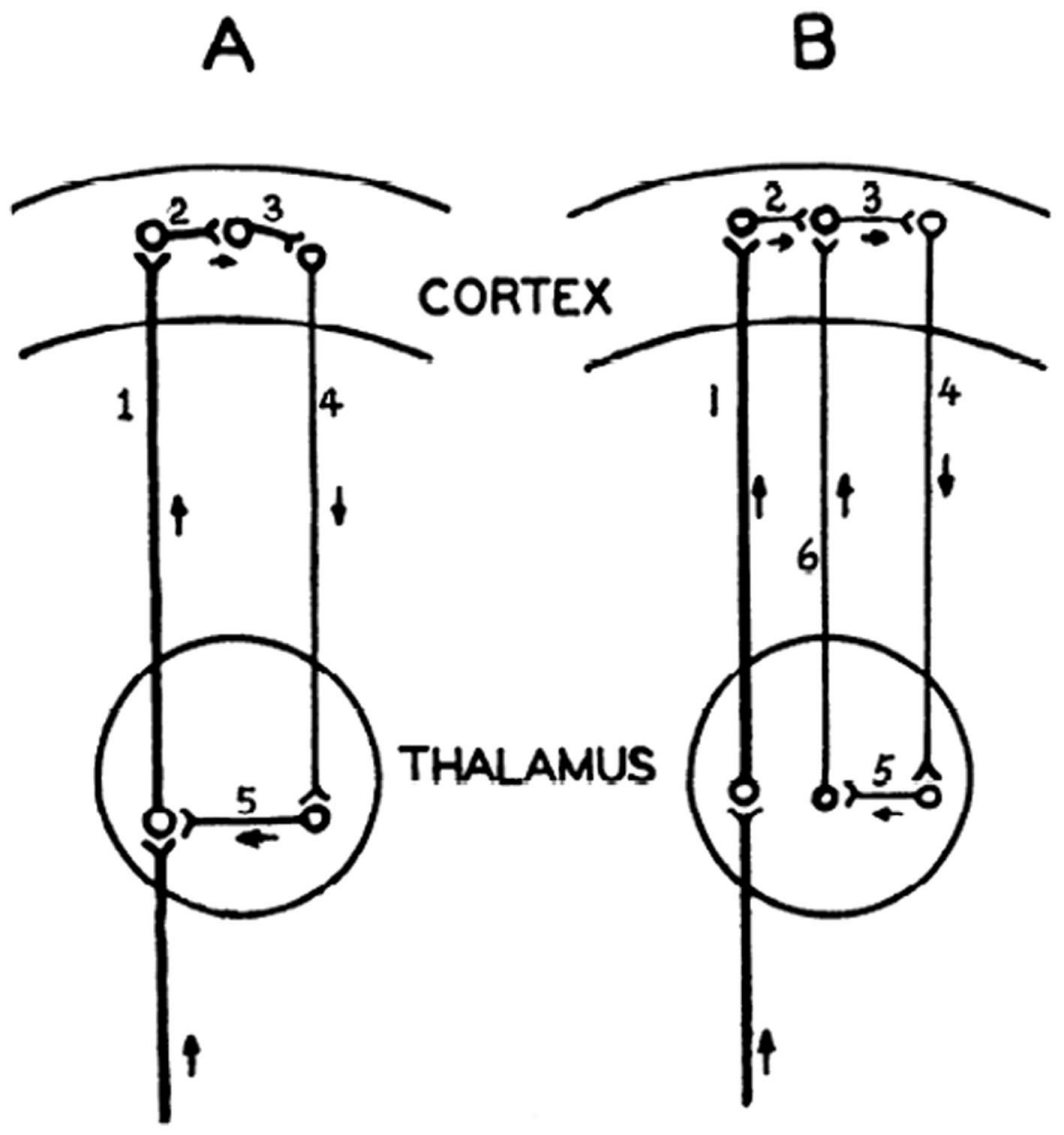
A diagram from Chang (1950, page 250) showing two possible pathways of the corticothalamic reverberating circuit. In **(A)** the corticipetal pathway of the reverberating circuit shares the same neurons which mediate the great afferent volleys. In **(B)** they do not use the same neurons. Diagram B is believed to be more probable. Chang (1950, pages 249–250) explains this diagram as follows: “The hypothetical pathway of the corticothalamic reverberating circuit is diagrammatically represented by a sketch in **B**, in which it is shown that the primary afferent volley and the repetitive discharges do not use the same thalamocortical neurons. One might question how the repetitive discharges to the first stimulus could be blocked or abolished by the primary response to the second stimulus, if it is the case that the primary response and the reverberating discharges do not use the same thalamocortical afferent pathway. This paradox will be readily understood when one makes a careful inspection of the scheme visualized in diagram B above. Suppose a volley of corticipetal impulses of a reverberating wave arrives at neuron 3 in the cortex by way of neuron 6 shortly after the arrival at the same neuron (neuron 3) of the second primary afferent volley from neurons 1 and 2; the reverberating impulses would find themselves falling into the refractory period of that neuron (neuron 3) left by the second primary response and they would therefore fail to go on propagating. According to this argument, the blockage of the reverberating waves by the preceding primary response apparently occurs in the cortical neurons rather than in the thalamocortical fibers. The supposition of a common ascending pathway between the cortex and the thalamus is not necessary for the interpretation of the blocking phenomenon in question.”

Chang (1950, page 255), who worked at Yale University, pointed out that the functional significance of the corticothalamic reverberating circuit (see Figure 11**)**, as suggested by Cajal, is that “these fibers constitute an anatomical basis of the mechanism of *sensory attention*, i.e., the capacity of limiting our conscious activity to a particular region of sensory fields, cutaneous, visual or auditory. Cajal’s conception was derived mainly from his anatomical observations made almost fifty years ago when the reverberating mechanism was not yet comprehended.” Chang (1950, p. 255) continued to say that: “From a functional point of view it is significant that by means of a corticothalamic reverberating mechanism a single volley of afferent impulse can set up a potential change in the brain outlasting by several thousand times the duration of the original stimulus. This mechanism seems to constitute an elementary physiological substratum for the formation and the persistence of a mental impression aroused by a sensory stimulus.” Thus, Cajal had understood the functional importance of reverberatory loops as early as 1904, but it was not until Lorente de Nó expanded on this work that their importance for cognitive functions of the brain was realized.

One important aspect of Cajal’s work is that he put arrows on his carefully drawn figures to show the direction of current flow (See Figures 9A,B). As stated by Sherrington (1949, p. xii), “Cajal made it possible even for a tyro [novice] to recognize at a glance the direction taken by the nerve-current in the living cell, and in a whole chain of nerve cells. He solved at a stroke the great question of the direction of the nerve-currents in their travel through the brain and spinal cord”. Cajal showed that there were recurrent circuits [feedback loops] in the cerebellar cortex (see Figures 9, 10). However, as pointed out above, the arrows showing the direction of impulses in Cajal’s diagrams were only hypothetical. Actual data on the direction of nerve impulses had to await the work of Lorente de Nó (1933).

Lorente de Nó began to work under Ramon y Cajal in Madrid in 1920, when he was an 18-year-old student and received his medical degree in 1923, at age 21. In 1924 he visited labs in Utrecht (Rudolf Magnus) and Leiden (Jan van der Hoeve) on his way to Uppsala to study physiology with Gustaf Göthlin in the institute of Robert Bárány, where he stayed for 3 years. From Oct 1926 to Feb 1927, Lorente de Nó made a research visit to the Kaiser Wilhelm Institute for Brain Research in Berlin where he worked on the anatomy of the medulla oblongata and the cytoarchitecture of Ammon’s Horn with Oscar and Cecile Vogt (see Lorente de Nó, 1938d, 1943). For a review of the work of the Vogts, see Nieuwenhuys (2013) and Amunts (2026). It was during this visit that he became an expert in using the camera lucida (Wollaston, 1807) and photomicrography to depict neurons and their connections (See Larriva-Sahd, 2002, 2014; Espinosa-Sanchez et al., 2020).

According to Larriva-Sahd (2014, page 5), Lorente de Nó was critical of the drawings made by Cajal and Golgi: “While Ramón y Cajal and Camilo Golgi performed their drawings from information kept in mind after observing the specimen, Lorente de Nó developed a variant of the Germanic approach. Researchers, particularly Oskar Vogt and his disciples, who were concerned with the proportions and dimensions of every layer or nuclei, utilized either camera lucida drawings or even photographic reproductions from large-format negatives. Lorente de Nó designed a hybrid system consisting of a projecting prism placed directly at the eye piece of the microscope. This straightforward procedure allowed him to focus the microscopic image directly onto the working-table; then, the image was accurately copied; ‘every drawing should be a replica of a neuron’, he said.”

Lorente de Nó (1922, page 6) states that he used the Edinger apparatus (described by Gage, 1912) to make his drawings. In two places, Lorente de Nó is quoted as saying that he refined this apparatus and published a paper on his refinement, but I could not locate either paper. First, Lorente de Nó (1922, page 6) writes that “the Cajal reduced silver method [will] be described in greater detail in a communication to appear in the *Zeitschrift für wissenschaftliche Mikroskopie*”, but a footnote by the translaters states that “there is no indication of the appearance of this communication in Lorente de Nó’s personal bibliography.”] Second, In an interview with Larriva-Sahd (2002, page 8) from his retirement home in Tuson, Arizona, Lorente de Nó is quoted as saying that “I designed a simple way to project microscopic images from the eyepiece to the table work. As a matter of fact, I early published the description of the device in Cajal’s journal (*Trabajos del Laboratorio de Investigaciones Biológicas*)”. But I have not been able to find this paper.

de Castro (2021) has reviewed the art of the drawings made by Cajal and his school (see also Junkes and Nardi, 2025) and noted that Lorente de Nó (1932) did publish a short description of his photo-micrographic apparatus.

Lorente de Nó expanded on Cajal’s concept of recurrent circuits in developing his studies on the nature of the reflex arc. For Lorente de Nó, the reflex arc was not “a unidirectional transmission system”, but “groups of neurons arranged in half-centers and capable of rhythmic activity”. Lorente de Nó (1933, p. 247) noted that “the existence of such complicated circuits has been disregarded”, and he spent 12 years examining the patterns of neural connections in great detail. This involved the study of interneurons and their role in neural circuits (Fairén, 2007) and resulted in the development of two laws describing the connections within chains of neurons: The Law of Plurality of Connections and The Law of Reciprocity of Connections [Lorente de Nó (1933); depicted in Figure 10]. It was these connections that Hebb (1944a) summarized in his unpublished notes on Lorente de Nó (see Appendix 1).

Fairén (2007, page 439) reviewed the reverberating circuits of Lorente de Nó (1933, 1938a, 1938b, 1939) and noted that “In the celebrated arrow diagrams of Cajal (see for instance Figure 6 in Sotelo, 2003; Figure 2B in Llinás, 2003) interneuronal connections are always feed-forward. Cajal has never considered feedback. Just with one exception, when he describes the connections between parallel fibers to Purkinje cells and to Golgi cells. As it is well known, Golgi cell axons contact the dendritic digits of granule cells at the periphery of the glomerule. This closed a reverberant circuit, although Cajal did not consider such a possibility in his discussion. Nevertheless, the existence of such circuits seemed to have posed a conceptual point of disagreement between Cajal and Lorente [de Nó].”

Fairén (2007, page 439) continued with a passage from Freeman (1984, page 233) which said that: “Lorente later recalled (personal communication) that in the mid-1920s he prepared a manuscript on the cytoarchitecture of the cerebral cortex, in which he concluded that feedback relations were a prominent feature. After reading the manuscript, Cajal strongly urged Lorente not to publish it because it would be unacceptable to the scientific community and might blight his career. Out of respect for his mentor, Lorente elected not to publish the material while Cajal lived, when he did publish (Lorente de Nó, 1934b), the work established itself as one of the enduring classics in neuroanatomy. Its influence on neuropsychology (through Donald Hebb), on neural modeling (through Warren McCulloch), and on computer design (through John von Neumann by way of McCulloch) has been incalculably great.”

The evidence for the quotation above from Fairén (2007) is as follows. Lorente de Nó published his first paper on the anatomy of the cerebral cortex of the mouse in 1922, and in his 1933 paper he first published his diagrams of chains of neurons representing his laws of the plurality of connections and the reciprocity of connections as further described in Lorente de Nó (1934b). Lorente de Nó (1933, page 248) said that “During the last 12 years I have been conducting studies on the structure of the nervous system with the purpose of determining whether the general plan of structure is represented by the unidirectional transmission system of [Cajal’s] Figure 1 (See Figure 9A), or rather by circuits similar to those in [Cajal’s] Figure 2 (see Figure 9B). To answer this question it was necessary to investigate, with the help of Golgi’s and Cox’s methods, the structure of the different nuclei and their connecting pathways and to determine whether or not cells with short axis-cylinders and recurrent collaterals or centrifugal fibers are present.” Thus, the gap between the 1922 and 1934 papers is explained by Lorente de Nó’s statement that he had been working on the problem for 12 years and by the biographical paper of Quiroga (1999) that describes Lorente de Nó’s disjointed career path during those 12 years before he moved to the USA.

Lorente de Nó (1939) did not believe in chemical synapses in the brain and his theories of neural function, based on electrophysiological studies, were focused on electrical synapses. As pointed out by Espinosa-Sanchez et al. (2020, page, 1227), “the neurophysiologists, including John Eccles, Gasser, Erlanger, and Lorente de Nó, maintained that impulse transmission was electric. By 1945, based on his own experiments, Eccles began to accept the chemical hypothesis (see Eccles, 1990). However, it was not until 1949 that Lorente de Nó finally accepted the chemical hypothesis.” So this may explain why Hebb focused on electrical synapses in his neuropsychological theory.

**Figure 12:**
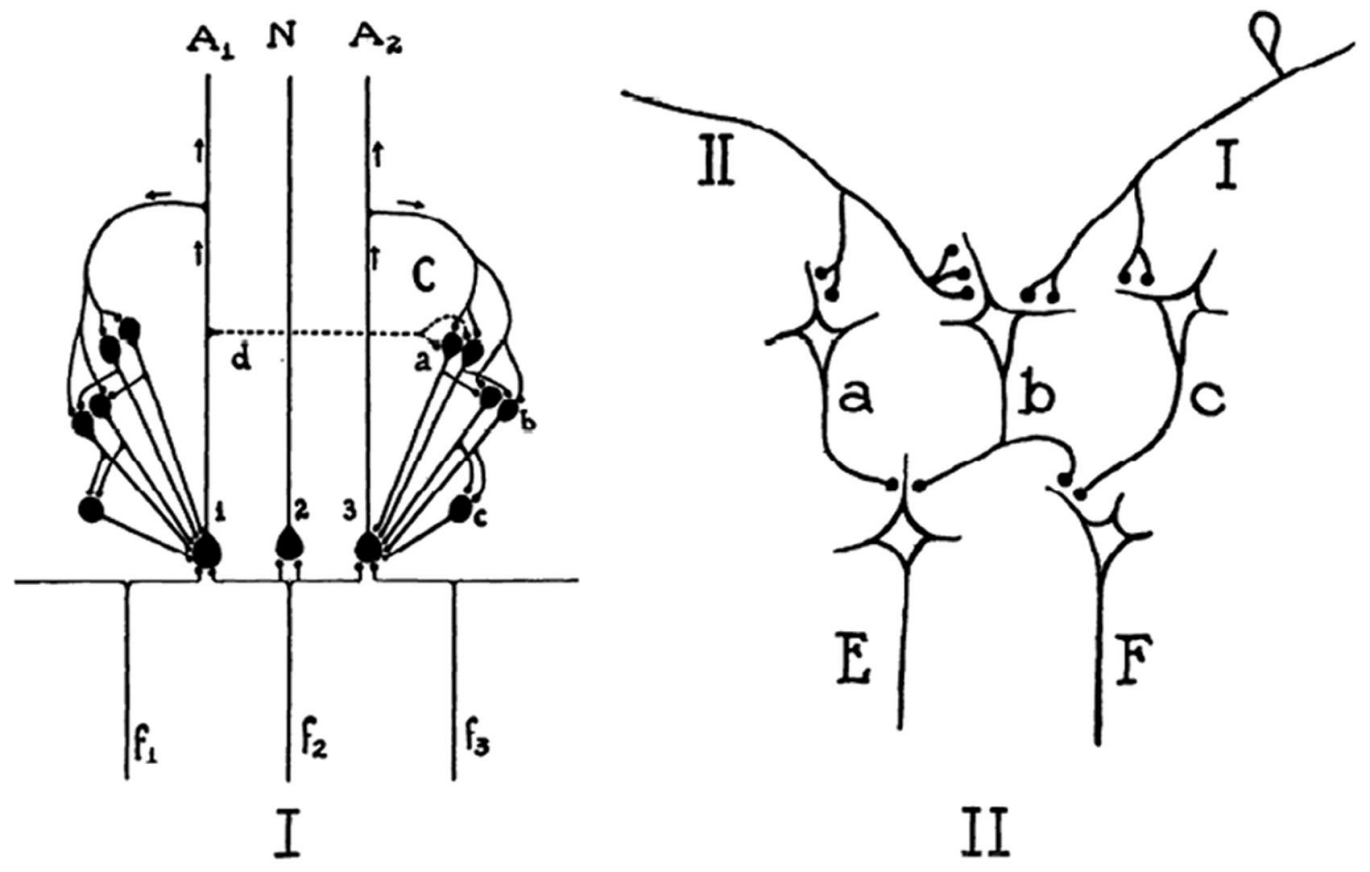
Lorente de Nó (1938c, page 231, Figure 12) depicted two ways in which inhibition might occur in neural circuits. (I) shows a diagram explaining the production of reflex reversal by concurrent stimulation of two fibers (*f*_1_ and *f*_2_ or *f*_2_ and *f*_3_) from different peripheral sense organs and its maintenance by the impulse conducted by closed chain *C*, after fiber *f*_3_, which initiated the response of cell 3, ceases conducting. Each one of the links in the closed chain represents a multiple chain of neurons (see *M*, Figure 10B). Collateral *d* by lowering the threshold of cell a and thus causing two impulses to cross through cell 3 in quick succession may produce inhibition, for cell 3 will acquire a high subnormal threshold. (II) shows a diagram from Gasser (1937) explaining reciprocal innervation. It is assumed that threshold stimulation of neurons with normal threshold requires simultaneous activation of two synaptic knobs, but three knobs are required to stimulate a neuron having subnormal threshold. When fiber I conducts a rhythmic series of impulses at low frequency neuron *F* is stimulated by the *b* and *c* impulses and the flexor muscle contracts, but if fiber II then becomes active, cell *b* will be forced to discharge an extra impulse and to acquire subnormal threshold. Henceforth cell *b* will be able to respond only to the impulses conducted by fiber II, the result being that the extensor muscle contracts, while the flexor muscle relaxes because the *b* and *c* impulses reach neuron *F* at intervals longer than the period of effective summation of impulses delivered at neighboring synapses. Lorente de Nó (1938c, page 232) noted that “a closed chain having a large number of links can remain in activity for considerable periods of time; but short chains, in which the impulses circulate at a high frequency, must have a short time constant, because the passage of only a few impulses will create a high threshold and stop conduction. Inhibition must then result. For example, in diagram I of this figure impulses may circulate through the closed chain C at different rates. If the circuit should be closed through cells *a* the rate would be high, because the impulse initiated in cell 3 would return in about one msec. to the same cell after crossing cells *a*; but if the cell 3 impulse should fail to reach the threshold of cells *a* and return through b, the rate would be lower. With a long chain, i.e., one with many internuncials in series, or a chain including long fiber paths, the rate of circulation may be low enough to allow activity for considerable periods of time, especially when fiber *f_2_* or *f_3_* also is conducting a series of impulses. However, if while chain C is working at low frequency, facilitation of cells *a* by impulses through collateral *d* should force the impulse from 3 to cross these cells and to restimulate cell 3, the activity must cease, because two discharges at the rate of 1,000 per second create in cell 3 so high a threshold that further transmission through this cell is blocked. Thus, the activity of closed chains of internuncial neurons leads, under certain conditions, to facilitation, and under other conditions to inhibition.

Lorente de Nó (1936, p. 244) concluded that since “the only evidence of inhibition is absence of response”, then inhibition can be satisfactorily explained in terms of lack of excitation.” Lorente de Nó (1938c) then developed a theory of inhibition based on neural circuits (see Figure 12). He believed (p. 238) “that specific inhibitory impulses, even if there were specific inhibitory fibers, do not exist. Inhibition must, therefore, be explained in terms of processes created by the same impulses that create excitation. The problem of inhibition has been submitted to careful analysis by Gasser (1937) who has suggested several diagrams accounting for different types of the phenomenon. Two elementary processes are considered: (1) rise in threshold of the neurons due to summation of subnormality; (2) lowering of the stimulating value of a synchronous volley of impulses by its fractionation into two volleys delivered at an interval longer than the effective period of summation of impulses arriving at different synapses.”

According to Lorente de Nó (1938c, page 232–233), inhibition could occur in closed loops in the following way: “Since the summation of subnormality progresses but slowly at low frequencies, a closed chain having a large number of links can remain in activity for considerable periods of time; but short chains, in which the impulses circulate at high frequency, must have a short time constant, because the passage of only a few impulses will create a high threshold and stop conduction. Inhibition must then result.” He argued that “the activity of closed chains of internuncial neurons leads, under certain conditions, to facilitation, and under other conditions to inhibition.” His final statement (page 241–242) was that “The closed chain of neurons (C, in Figure 10B of the present paper) may play different roles according to the number of links that it contains. If the number is small, activation of the chain may result in inhibition, but if the number of links is large enough it may result in sustained facilitation or discharge” (see Figure 12).

It was the line drawings of the cells and recurrent pathways in neural networks describing the Law of Reciprocity of Connections of Lorente de Nó (1933, 1938c) on which Hebb’s (1944a) unpublished notes were focused (see Appendix 1). As noted above, before the work of Cajal and Lorente de Nó, the concept of a reflex was a linear connection between pre-and post-synaptic neurons. The concepts of interneurons, and the existence of complex multiple and closed circuits shown by Lorente de Nó (1938c, 1938d) meant that reflexes were far more complex than previously thought.

Lorente de Nó (1938b) focused on the role of internuncial neurons and synaptic knobs in activating excitatory responses in motor neurons. Hebb (1944a) was interested in the complexity of the synaptic connections described by Lorente de Nó, as shown in Figure 6 [which Hebb originally drew by hand in1947 and then copied and used as Figure 6 in his 1949 book]. An important point in this paper for Hebb’s theory was the statement that “Although threshold stimulation of motoneurons requires that several impulses be delivered simultaneously at their synapses, not all the fibers contributing to the synaptic scale need be active” (Lorente de Nó, 1938b, p. 205). However, for Hebb, the most important paper was Lorente de Nó (1938c) in which the two types of chains formed by internuncial cells were described: multiple (M) and closed (C) chains. The closed chains demonstrated recurrent loops, also shown as Figure 66 in Lorente de Nó, 1938d (See Figure 10).

When discussing inhibition, Lorente de Nó (1938c, pages 238–241) stated that “At present it is generally believed that specific inhibitory impulses, even if there were specific inhibitory fibers, do not exist. Inhibition must, therefore, be explained in terms of processes created by the same impulses that create excitation” (p. 238) Inhibition was therefore considered as “a deficit of response” and thus the idea of reciprocal inhibition was given prominence (see Figure 12). Hebb’s (1934) discussion of reciprocal inhibition and his drawing (see Figure 4), based on Sherrington (1925) were in agreement with the discussion of Lorente de Nó (1938c), even if Hebb did not read this paper until 1944. It is notable that Lorente de Nó (1938c, pages 232–234) includes a discussion of “long-lasting facilitation” which appears to predate the discovery of long-lasting facilitation by Bliss and Lomo (1973), later termed Long Term Potentiation by Douglas and Goddard (1975).

As noted in Brown (2020), Lorente de Nó’s ideas on inhibition had a significant influence on Hebb. Lorente de Nó (1938c) described inhibition in a closed loop and Morgan (1943, pages 66–67) described successive inhibition and reciprocal inhibition. In Hebb’s (1944b) unpublished notes (see Appendix 2, page 5) he said “I am deliberately vague about when or where one LNC will (A) facilitate another, and hence make possible structural (B) facilitation; simultaneous activity not enough, because of (1) anatomic separations, (2) apparent fact that one system can inhibit or extinguish another, (3) factors of timing utilized by Lashley in his theory of repetitive circuits”. However, when Hebb (1945b) wrote his Precis (see Appendix 5), the idea of inhibition was dropped. Although Hebb (1949a) referred to Sherrington’s (1925) central inhibitory state (c.i.s.), he did not consider inhibitory synapses. Hebb (1949a, page 214) considered inhibition as fatigue of excitatory pathways and stated that “If it were safe to assume a true long-term inhibition (or equally, fatigue) in cerebral action it would greatly simplify the task of the psychologist. As matters stand, however, this is not justified.” According to Fairén (2007, page 436), Lorente de Nó “conceived the existence of inhibition, and wrote that internuncial neurons might be the siege of it, then he disdained this concept absolutely, and did not mention it further”. From this discussion, it seems that Hebb followed the lead of Lorente de Nó and thus did not include inhibition in his neuropsychological theory.

## 7 Hebb’s unpublished notes for his neuropsychological theory: 1944–1946

In planning his theory between 1944 and 1946, Hebb made a series of hand-written and typed notes and the McGill University archives contain five sets of these notes that he made while organizing his thoughts on the neural basis of behavior: (1) Hebb’s (1944a) notes on Lorente de Nó ‘s papers dated February 1944 (Appendix 1); (2) Hebb’s (1944b) [incomplete] concept speculations dated about June 1944 (Appendix 2); (3) Hebb’s (1945c) Structure and origin of concept speculations written in spring ‘45 (Appendix 4); (4) Hebb’s (1945d) Precis: The structure of a set of neuropsychological speculations dated March–July ‘45 (Appendix 5); and also (5) Hebb’s (1946c) copy of “most of the original MS of *The Organization of Behavior*”.

In his unpublished notes for planning his neuropsychological theory between 1944 and 1946, Hebb used the concept of a schema in two ways. First, in his June 1944 notes (Appendix 1), Hebb (1944b) refers to the schema of a rat learning to find food in one of the four corners of a table as described in his papers on field orientation (Hebb, 1938c; Hebb, 1938b). This seems to refer to a plan, a concept or a sequence of motor acts which would provide a memory for the location of food on the open field table and the series of motor acts required to obtain the food. In his “concept speculations” Hebb (1944b) linked his schema for maze learning by the rat with Lorente de Nó’s reverberating circuits and provided his first thoughts on the cell assembly (LNC’s) and the phase sequence (see Appendix 2). However, in his *Structure and origin of concept speculations,* and his Precis Hebb (1945d); Hebb (1945c) used the term “schema” to describe his neuropsychological theory, the aim of which was to explain how psychological concepts could be understood in physiological terms (see Appendix 4, 5). This schema was developed in his 1945 notes (Hebb, 1945a, 1945b), his paper on the nature of fear (Hebb, 1946b) and in his first draft of *The Organization of Behavior* (Hebb, 1946c). Before Hebb defined the term “Cell Assembly,” he used the term “Lattice”, and he called this idea his “schema”, thus it seems that in these notes, Hebb used the terms schema and schemata to mean a pattern of integrated nervous activity.

Rumelhart (1984, p. 162–3) argues that schemata are “the foundational elements upon which all information processing depends” and defines a “schema theory” as “a theory about how knowledge is represented and about how that that representation facilitates the *use* of the knowledge in particular ways”. He uses the concept of a schemata to refer to (1) a memory system or (2) a way of describing a theoretical system in which the components are unknown, which describes Hebb’s use of the term ‘schema’. At first, Hebb had no name for his theory. He called it a schema; what Rumelhart (1984, p. 166) terms “a kind of informal private unarticulated theory”. Second, Hebb’s schema was an active process which involved procedures of synaptic change, recurrent circuits and connections of neural networks involving ‘sub-schemas’ connected to form phase sequences. Thus, as defined by Rumelhart (1984, p. 167), Hebb’s cell assembly schemata was an active process with “a very well defined constituent structure” that could be “viewed as a procedure whose function is to determine whether, and to what degree, it accounts for a pattern of observations” related to the neural control of behavior.

In a set of notes dated Feb. 1944 (Appendix 1), Hebb (1944a) summarized the papers of Lorente de Nó (1933) and Lorente de Nó, (1938c) and Lorente de Nó (1938b). In these notes on Lorente de Nó (1933) he paid attention to the Law of Plurality of connections and the law of Reciprocity of connections (see Figure 10 of the present paper). It is noteworthy that Hebb’s final note on these papers was that “Somewhere in this mess is the secret of inhibition”.

In these notes, Hebb was particularly drawn to Figures 3–5 in Lorente de Nó (1938b) and he reproduced Figure 3 in Chapter 4 (page 64 of *The Organization of Behavior*, shown here as Figure 6). It is clear that Hebb’s thinking was influenced by Lorente de Nó’s work as he says that: “Lorente de Nó (1938b, page 198) has shown that the synaptic knob is usually not a terminal structure (thus the term “end foot” or “end button” is misleading), nor always separated by a stalk from the axon or axon collateral. If it were, of course, some action at a distance would be inevitably suggested, if such connections are formed in learning. The knob instead is often a rather irregular thickening in the unmyelinated part of an axon near its ending, where it is threading its way through a thicket of dendrites and cell bodies” (Hebb, 1949a, p. 65). Since there were no electron microscopes in 1938, the synapses appeared as a dark blobs in the microscopes of the time (See Figure 6). In the final part of Hebb’s, 1944a notes, he examined the figures in Lorente de Nó (1938b, pages 200, 201 and 203). The figure on page 200 (Lorente de Nó, 1938b; Figure 3, became Hebb, 1949a, Figure 6, [see Figure 6]) and Hebb made a number of notes on this figure, which he printed on page 65 of *The Organization of Behavior.* The final section of Hebb’s (1944a) notes says that: “He (Lorente de Nó) believes that stimulation, crossing of synapse, requires activation of all knobs in small area” (see Appendix 1).

Hebb was so impressed with the work of Lorente de Nó, that he wrote to him on 28 April 1944 “to find out whether you would consent to my coming into your laboratory, for perhaps a month, at some time during the coming summer or autumn” (see Appendix 3). Hebb’s goal was to learn some neurophysiology in Lorente de Nó’s laboratory because, as he stated in his letter “in the field of neurophysiology in general my ignorance offers a real barrier to understanding much published work”. On 1 May 1944, Lorente de Nó wrote back to Hebb to say that “At present, my work is concerned with the relationship between the production of the nerve impulse and the metabolism of nerve, a problem that is of little immediate interest to a psychologist.”

On 4 May 1944, Hebb wrote back to Lorente de Nó to say that “As you perceived, it was your work on the synapse of which I was thinking in writing to you. Your suggestion that another time might be more suitable for a visit to your laboratory seems wise, therefore, and I shall make other plans (Appendix 3).” It is notable that Hebb included in this letter the statement that “I am profoundly convinced that psychological progress will depend on research such as yours—not that psychologists should necessarily do such research, for there are problems at the level of gross behavior which apparently must be studied as such, but that psychological theory can go no farther without a more detailed basis in neurophysiological fact.” On 26 April 1949, Hebb again wrote to Lorente de Nó for permission to use the figure from Lorente de Nó (1938b, p. 200) in *The Organization of Behavior.* He ended this letter with the statement that “I believe that my book will be able to show that modern ideas in neurophysiology, and particularly some of those that you have developed, have a revolutionary significance for psychological theory” (see Appendix 3).

The first three pages of Hebb’s (1944b) Concept Speculations notes (dated “about June ‘44″) are missing from my copy, which begins with the notation “4 concept speculations—outline for 3rd presentation, following 5(g), with preliminary references to parts already dealt with” (see Appendix 2). So there must be a set of notes before these, consisting of 3 pages entitled: “1 Concept speculations, 2 Concept speculations and 3 Concept speculations,” which have concepts 1 to 5(g). They may have been notes for a presentation to his colleagues at Orange Park.

In these notes, Hebb is trying to develop a schema, or theory, of the neural control of behavior. Initially (in his 1932 MA thesis and 1934 Chicago paper) his theory revolved around the concept of the simple reflex and the problem of inhibition, but he did not get very far. As soon as he was aware of the work of Lorente de Nó, he realized that reflexes involved complex interactions among neurons with M and C connections. In these notes, Hebb (1944b) is trying to combine Lashley’s ideas with the recurrent circuits of Lorente de Nó to develop his schemata for the neural basis of behavior. He refers to Lashley 3 times: first to the idea of innate organization (in A); then to the idea that rat learning in a maze is not a function of any one sense mode (in B); and then to the jumping stand (f). In these notes, Hebb (1944b) also tried to integrate his ideas on the concept of ‘set’, the definition of ‘organization’, perceptual learning, and the ACP (autonomous central process), with his understanding of Lorente de Nó circuits (LNCs). He also examined the problems with Gestalt theory, the role of attention in learning, and the results of his Chicago research on maze learning rats in the open field in developing his schema to account for the neural basis of behavior. In these notes, Hebb mentioned the concept of Lorente de Nó circuits (LNCs) five times to account for the neural basis of these behaviors. The complete set of these notes is in Appendix 2 and here I note only the sections which include Hebb’s mention of the LNCs. These notes were for Hebb’s use only, so consist of disconnected jottings. I copy them here to illustrate his thought processes:

(5 g. B) What I mean by organization behaviorally: ready identification or discrimination, ease of recall and accuracy of recall or identification, ease of association in any circumstances and physiologically: assumption of need of a number of parallel LNCs (Lorente de Nó Circuits) for stable response or perception, possibly of good organization by temporary facilitations, possibly of good organization by innate physiological and anatomical factors, possibly of organization by structural modifications.

(5 g. B, con’t). Idea that space perception fundamental in learning: evidence from rat, very brief, and even in chimp, from his greater ease of associating place and holding it in mind (delayed response evidence), or man (number forms, etc.; not need to be conscious even if all have them; if numbers learned on space coordinates, superimposed LNC activity would modify the total enough so that we might suppose it only by accident that one would be conscious of space-coordinate basis of identification of numbers, etc.)

(6). Mechanism of Learning. (6a) Learning already referred to, here deal with learning of actual habits, instead of perceptual learning: return to assumption (6)—facilitation A, transient, facilitation B more stable; possibility of one-trial learning if A lasts long enough to establish B (about whose time requirements we know nothing.)

(6b) I am deliberately vague about when or where one LNC will (A) facilitate another, and hence make possible structural (B) facilitation; simultaneous activity not enough, because of (1) anatomic separations, (2) apparent fact that one system can inhibit or extinguish another (Dusser de Barenne and McCulloch, 1939), (3) factors of timing utilized by Lashley (1937, 1938) in his theory of repetitive circuits. Idea of diffuse engram makes possible unimportance of anatomical factors, but facts of aphasia, selective effects of different locus injury in man and animal suggests difference, grossly determined, in locus of various LNCs, so this presumably operates.

(6j) Establishment of a learned response, as consistent and predictable, presumably involves the activation of a minimal number of LNCs; early in learning, before much structural change, a larger number necessary than afterward; the facts of attention seem to suggest that there is a central pool of LNCs which can selectively facilitate sensory activities (ACP as already urged) and also that the pool is essential to a new complex learned act; if barely within the subject’s p[o]wers, always necessary; with time, and a relatively easy act, not necessarily, and is “freed” from the act. The mechanism of freeing I think is found in the involvement of the same pool in new acts, which set up new relationships within it. The pool is, I think, the basis of voluntary action as well as of thought.

This concludes the sections which directly refer to LNCs in Hebb’s (1944b) notes (see Appendix 2).

In his notes entitled “Structure and origin of concept speculations” Hebb (1945c) focuses on perceptual learning, the role of ACA (Autonomous Central Actions) in sensory and motor processes and the role of LNCs (Lorente de Nó Circuits) in these processes (Appendix 4). This hand-written 4-page set of notes seems to have been stimulated by Lashley’s paper on “functional determinants” (Lashley, 1937). In point A(6), Hebb (1945c, page 1) says “That perception is certainly not as anatomically unified as it appears to be—not until I developed this idea did I realize the force of KSL’s paper on “functional determinants.” Later Hebb links the ideas of Lashley (1937) with those of Lorente de Nó, proposing that Lorente de Nó circuit activity might provide an anatomical basis for perceptual generalization. In the “functional determinants” paper, Lashley (1937) argued against the concept of “Localization of Function” in the cortex and suggested that different cognitive functions such as spatial representation, timing and the serial order of behavior are controlled by different brain areas which are integrated into a “pattern” of dynamic neural processes. Lashley (1937, p. 386) stated that: “The position of Goldstein [Kurt Goldstein 1934. *The Organism*. The Hague, Netherlands] that the functions of every center are dependent on its relations to the rest of the intact nervous system, cannot be too strongly emphasized in considering problems of neuropsychology.” Hebb (1945c) thus obtained two important concepts from Lashley’s (1937) paper: (1) that cognitive functions involve the integrated interactions of a number of different cortical locations, and (2) that the recurrent circuits of Lorente de Nó (1933, 1938c) are “especially adapted” for this integrative function.

In this typed 45 page “Precis: The structure of a set of neuropsychological speculations” of March–July ‘45 (Appendix 5), Hebb (1945d, page 1) explains the schema for his planned book: “The introduction (A) deals with aim, scope and mode of approach, from the point of view of a possible writing up and presents a fundamental assumption concerning the nature of cerebral action. Part I. (B) to (F) tries to establish the probable truth of a series of propositions concerning perception. (G) then makes a bald assumption concerning the nature of learning, and (H) tries to show that learning is in terms of previous learning—that new habits “utilize” instead of merely replacing older habits. From this point on, the attempt is made to show that a schema of the physiological control of behavior can be developed by utilizing these preliminary ideas, and, from the schema, to develop conceptions of value to the analysis of behavior.”

Hebb (1945d) continues to say that: “The schema does not make specific predictions and thus suggests only a reformulation of the problem of the control of behavior, instead of providing an explanation. There is an approach to explanation, however, in the fact that it shows how a variety of psychological problems may possibly be unified, and how it is possible for example to conceive of a mechanism of the effect of attention on learning, in physiological terms; and suggests that if the hurdle of perceptual generalization can be got over, the problem of motor generalization and its perceptual control (as discussed by Lashley, 1937) becomes physiologically explicable (in general terms, of course, and for simple cases). The system is not merely a translation into physiological terms, therefore, but does suggest the basis of a considerable synthesis and makes possible an intelligible formulation of the problems of attention and thought, as a function of cerebral processes.”

In Proposition G of this Precis, Hebb (1945d, pages 15–21) relates in some detail how the results of Lorente de Nó influenced the development of his “schema” (see Appendix 5). Proposition G begins with an introductory statement which states that: “Without any direct experimental evidence to support it, the assumption is made that lasting structural changes occur to reinforce and perpetuate the temporary, dynamic facilitation whose existence is a necessary inference from Lorente de Nó’s work. This makes the assumption that there is a dual mechanism of the trace, a “dynamic” plus a “structural” engram.” (p. 15).

After discussing Hilgard and Marquis (1940, p. 331) summary of Lorente de Nó’s conclusions in proposition G1, Hebb (in proposition G2) gives the first definition of what has become known as the “Hebb synapse”: “The assumption is consequently made that long persistence or repetition of the dynamic facilitation (of (G1) above) tends to induce structural changes which reinforce the facilitating action. The assumption can be put as follows: when cell A is in propinquity with cell B and repeatedly or persistently takes part in firing B, some growth process or physiological change takes place in one or both cells such that A’s efficacy as one of the cells firing B, is increased.” (p. 16).

The discussions in sections G3 to G7 expand upon the importance of Lorente de Nó’s ideas to Hebb’s schema, and how Hebb plans to deal with the development and possible critiques of his schema. Rather than reprint them here, I attach Hebb’s notes for section G in Appendix 5. These concern Lorente de Nó’s (1938, p. 198) suggestions concerning the development of “synaptic knobs,” and their possible facilitation of long-term memory; the type of structural changes at synapses that might underlie memories; the difficulties in the mechanism through which one reverberating system might facilitate another; the fact that some reverberatory systems could be facilitated while others are eliminated; and, finally, in section G7, Hebb’s comments on the fact that Lashley was critical of the theory of synaptic change underlying learning and memory (see Lashley 1934). Section G6 ends with the following statement: “In general, it can be said that the consequences of Lorente de Nó’s demonstration that the synapse is crossed only by the simultaneous action of two or more impulses has a much greater significance for psychological theory than appears on the surface. The connections postulated here are between single cells, but the single cell is not the unit of excitation, and it is to a great extent by reason of this fact that the postulate avoids the rigidity of older ideas of “synaptic block” and “route formation.” (p. 21).

This ‘Precis’ is a preliminary draft of the first four chapters of *The Organization of Behavior*, which emphasize perceptual learning with a focus on the work of von Senden (1932) and the results of Lashley’s work on visual perception in rats. Figure 1 of this precis is Figure 4 (page 52) of *The Organization of Behavior*. The second part, entitled “Section V. A schema of perception” (pages 27–49) describes the development of “a schema of perception and perceptual learning,” some of which found its way into Chapter 5 of *The Organization of Behavior* (pages 83–99, including Figure 11) and some of which discussed the role of perceptual schemas in maze learning and motivation. Section V also discusses the role of Lorente de Nó circuits in perceptual learning. It is in this section that Hebb discusses “phases of the pattern of excitation” (p. 39), which he then termed a “phase sequence” (p. 40) and discussed in terms of perceptual learning (pages 40–43) based on his field orientation studies (Hebb, 1938a, 1938b).

At the same time that Hebb (1945b) was writing his Precis, he was also writing his paper on the nature of fear (Hebb, 1946b) in which he used his schema to explain the neural basis of fear. In this paper Hebb (1946b, p. 269) published his concept of a “phase sequence” for the first time: “It has already been seen that sensory and central processes contribute separately to the control of behavior. For convenience, let us designate the specific pattern of cellular activity throughout the thalamo-cortical system, at any one moment, as a ‘phase’. Behavior is directly correlated with a phase sequence which is temporally organized (Beach, 1942), in part by the inherent properties of the system (the constitutional factor) and in part by the time relations of various afferent excitations in the past (the factor of experience). The spatial organization of each phase, the actual anatomical pattern of cells which are active at any moment, would be affected by the present afferent excitation also. Subjectively, the phase sequence would be identified with the train of thought and perception. Now each phase is determined by a neural interaction, between the preceding phase and the concurrent afferent excitations. Lorente de Nó’s (1939) discussion of the dynamics of neural action shows that two or more simultaneous neural events might reinforce each other’s effects and contribute to a single, determinate pattern of subsequent cerebral activity; or on the contrary might be indeterminate, in the sense that slight changes of timing and intensity could lead to marked and sudden fluctuations of pattern. A phase sequence, that is, could be stable or unstable, and one can assume that vacillating, unpredictable and incoordinated behavior is the expression of unstable cerebral activity.” Thus by 1945 Hebb had all of the components of his ‘schema’ annotated and he began to work on a first draft of his book.

## 8 Completing the Organization of Behavior

The first draft of *The Organization of Behavior* was typed by Hebb (1946c) and my copy consists of about 101 pages, representing the first 5 chapters of the book. The outline lists 11 chapters and more than 248 pages, but I do not have a copy of final 6 chapters, which were written while Hebb was at McGill University in 1947–48. In my version, there are two chapters describing his schema. Chapter IV “Schema: The first stage of perceptual learning” uses the term “lattice” for what was later called the cell assembly, and Chapter V “The perception of a complex” describes the phase sequence. The references to Lorente de Nó in these two chapters are almost the same as in the published book (Hebb, 1949a). For example, the section entitled “The possibility of a dual trace mechanism” that refers to Lorente de Nó on page 58 of this draft (Hebb, 1946c) is almost word-for-word as the same section on page 61 of the published book. The reference to Lorente de Nó’s ‘synaptic knob’ on page 62 of Hebb (1946c) is the same as on page 65–66 of Hebb (1949a). Likewise, the discussion of Lorente de Nó’s contribution to the idea of a lattice on page 69–70 of Hebb, 1946c, including Figure 7, entitled “arrows represent a simple lattice of neural pathways) is the same as that published on page 73 of Hebb, 1949a, including Figure 10, entitled “Arrows represent a simple ‘assembly’ of neural pathways.”

As discussed in Brown (2006, 2020), Hebb sent a copy of his preliminary manuscript (94 pages) to Lashley, who responded with two pages of comments in February 1947, but was not very impressed. In a letter written to Henry Nissen on 19 May 1948, Hebb wrote: “he told me you know that the whole thing was very weak, with no value because it was so vague.” Hebb completed writing *The Organization of Behavior* at McGill University between 1947 and 1949 and received comments on how to improve his manuscript from Henry Nissen, E. G. Boring, Austen Riesen, Robert Blum and others.

On 29 September 1948, Hebb mailed the final manuscript to Thomas publishers under the title “On Thought and Behavior,” but Thomas returned Hebb’s manuscript because he had a number of other books to publish and so with the urging from Frank Beach and Henry Nissen, John Wiley & Sons agreed to publish Hebb’s book with the title *The Organization of Behavior* in the fall of 1949. As noted earlier (Brown, 2006, 2007) the reviews of *The Organization of Behavior* by Kuhn (1950), Brogden (1950), and Attneave (1950) were uniformly positive. Two critical reviews which are worth reading today are those by Leeper (1950) who critiqued Hebb’s contribution to learning theory, and Allport (1955) who examined Hebb’s theory of perceptual learning. Hebb (1963) again mentioned the importance of the work of Lorente de Nó to the development of his neuropsychological theory and answered some of his critics, particularly with reference to perceptual learning.

Hebb’s cell assembly theory has provided an important stimulus for understanding the neurophysiological basis of behavior (See memoirs by Bliss and Cooke, 2011; Hubel and Wiesel, 1998; McNaughton, 2025) and, as discussed by Posner and Rothbart (2004), Hebb’s ideas provide the basis for an integration of the disparate sub-fields of psychology. We have examined the origins of *The Organization of Behavior* (Brown, 2020; Brown and Milner, 2003) and Cooper (2005) provides a history and commentary on the Hebb synapse and learning rule. Hebb’s cell assembly theory attracted critical analysis shortly after it was published (Olds, 1954; Milner, 1957; Hebb 1959b) and the first computer programs designed to model the neural network activity of the brain were based on Hebb’s theory of synaptic change underlying the cell assembly (Rochester et al., 1956; George, 1963; Allport, 1955; White, 1961; Reitman et al., 1964; see reviews by Valentine, 1989 and Kuriscak et al., 2015), as are modern neuromorphic computer models of brain activity, including one called “Hebbnet” (Gupta et al., 2021; Chakraverty et al., 2019; Lin et al., 2025; Hua et al., 2026). The continuing importance of the book is discussed by Brown (2026); Brown (2020); and Brown (2017).

## 9 Discussion and conclusions

In this paper I have provided an outline of Donald O. Hebb’s career and how he developed the neuropsychological theory that was published in *The Organization of Behavior*. In his earliest writing on the neural basis of behavior in his MA thesis, Hebb (1932) stated that his purpose was “to present a theory of the functioning of the synapse based on the experimental work of Sherrington and Pavlov, on reflexes and inhibition.” As his theory developed, there was an increased focus on the role of the synapse and synaptic change in perception, learning and cognitive development, while the concept of inhibition, which was central to this thesis, was dropped from Hebb’s (1949a) final theory and had to wait for Milner (1957) revisions. Throughout this paper I have examined why Hebb omitted theorizing about the role of inhibition in neural function. Between 1932 and 1934, when Hebb was a PhD student, he struggled to develop a theory of the neural basis of behavior, but it was only after his discovery of the importance of the reverberatory loops of Cajal and Lorente de Nó that he began to develop his thoughts about the “Hebb synapse,” cell assembly and phase sequence that formed the basis of his neuropsychological theory.

Hebb stated that “It was in February 1944 that I came back to the problem of thought and the brain, when I found out that Rafael Lorente de Nó had recently shown that reentrant or closed circuits were to be found throughout the brain.” In this paper I have used the unpublished papers of Hebb from 1932 to 1934 to show his early thinking about developing a neurophysiological theory of cognitive function and then his unpublished notes and papers from 1944 to1946 which show how the ideas and the drawings of Lorente de Nó stimulated him to revise his conception of the neurophysiological basis of behavior. The excerpts from these unpublished notes and papers show how Hebb revised his early 1930’s theory between 1944 and 1946 and give some indication of the concepts which were retained in the final book and those that were deleted. These unpublished notes and papers show how Hebb’s thoughts developed as he revised his previous ideas on neural function and focused on integrating his own previous work, the papers of Lashley and the reverberating circuits of Cajal and Lorente de Nó into a coherent theory. The value of a paper such as this is to show the work that Hebb went through to construct his neuropsychological theory. These notes provide the background for Hebb’s (1959a, 1980a) autobiographical descriptions of how he developed the ideas that formed *The Organization of Behavior.*

## Data Availability

The original contributions presented in the study are included in the article/Supplementary material, further inquiries can be directed to the corresponding author.
