# Supplementary material for: The influence of Cajal and Lorente de Nó on Donald O. Hebb’s neuropsychological theory
Source: Front Neuroanat. 2026 Jul 27;20:1861011. doi: 10.3389/fnana.2026.1861011 (PMC13454044; doi:10.3389/fnana.2026.1861011)
Supplement: Supplementary file 1 [file Supplementary_file_1.pdf]

The influence of Cajal and Lorente de Nó on Donald O. Hebb's neuropsychological theory

Richard E. Brown

Appendix 1.

Hebb (1944a) Notes on Lorente de Nó. (1933 and 1938c)

LORENTE DE NO

date? Feb 1944?

Vestibulo-ocular reflex arc. (1) Arch. Neurol. Psychiat., 1933, 30, 245-291.

1. Cajal's idea, still in text books, of linear conduction over series of 2-3 fibres, as elementary unit.

2. Anatomically superimposed parallel circuits; and "long-circuits" of Fulton. Forbes.

3. Actually, no one has shown that this simple, ~~direct~~ linear circuit can function alone, and Graham Brown concluded that groups of neurons must be units, capable of rhythmic activity: and in the vestibular system, ~~the~~ nystagmus can be obtained when the main, direct connections of vestibular nuclei + oculo-motor nuclei are cut (fasciculus longitudinalis medialis); + nature of after discharge should change if really due to long circuits - also, it lasts too long.

4. Electrical phenomena occur in the cord in simple reflexes which cannot be explained by simple 2 or 3-neuron circuit (Barron + Matthews, 1936 - initiation of motor response either by slow rise of potential, or only after this). (e.g. - paper not seen):

5. Anatomical facts in themselves strongly against - page ~~1~~ 4

n+1 cell from n + n+1 - then n+2 — LAW OF PLURALITY OF CONNECTIONS - General

LAW OF RECIPROCITY OF CONNECTION - 2 exceptions: spinal sensory ganglia receive no afferents, apparently; + motor ~~sensory~~ cranial cells send out no collaterals.

6. Illustration with trigeminal - figure.

no way of knowing anatomically which is effective path - if anatomically shortest, no "use" for others - this kind of argument prominent (1933).

7. Vestibular reflex - smallest set of connections which produce 1 pair of nystagmus reflexes (figure)

General finding of system whose functioning is modified (~~decreased~~ in latency, ~~prolonged or reduced~~ after discharge, linear, etc) by any lesion - figure

Reticular system important = non-ordered complex, but more effective here than direct route alone

[Somewhere in this mess is the secret of inhibition

LORENTE DE NÓ

(2) Synoptic stimulation of motoneurons as a local process. J. Neurophysiol., 1938, 1, 195-206. Hebb 1938a

1. Direct stimulation of medial longitudinal fasciculus
   a. in mild ether narcosis (leaving corneal reflex intact) no effect
   b. although stimulation of reticular substance anterior to motor nucleus had
   c- and double stimulation of fasciculus had.
   d- No effect if vestibular nuclei + ret. nuc. in medulla destroyed
   e. - or if previous shock has suppressed, due to refractory state, background of normal internuncial bombardment.

2. Anatomy. Figure p. 200, 201, 203.
anterior horn, cat, 15 days; ventral acoustic ganglion of cat, 20 days; - 3rd is diagrammatic

<u>p. 200</u> fibre 6 synaptic junctions with 2 cells + a dendrite of another - <u>not</u> <u>end</u> buttons - "synaptic knobs"

large no. of knobs on <u>C</u>, but not close together: just as dev or <u>B</u> (or <u>d</u>) - interstices filled presumably with knobs of other fibres - next figure.

<u>2a</u> on dendrite - <u>2b</u> on C (+ d)
for <u>C</u>, fibres 2 + 6 in true relation = plane of section right.
+ partly for fibre 7, cell D - knobs in contact, fibres not.

cell I, presumably internuncial, from another section, shows the inter-relations - from any one fibre, knobs scattered - others interspersed - <u>even here, of course, many fibres not stained</u>

page 201, cell not stained, "an important part" of synaptic scale is - almost complete at dendrite <u>d</u>

L. de Nó, J. Neurophysiol. 1938, 1, 195-206 — (2)

normal state of affairs in intercellular spaces is,

(1) glia cells, blood vessels, and myelinated fibres

(2) plexus of dendritic branches; and

(3) un myelinated fibres with "numerous synaptic thickenings."

for (2), see fibres 17 + 18 p. 200 (figure)

for (3), see figure p. 208 (L. de Nó, J. Neurophysiol., 1938, 207-244.) Hebb 1938b

both (2) + (3) make numerous synaptic junctions.

He believes that stimulation, crossing of synapse, requires activation of all knobs in small area.

From other papers: limits of synaptic delay, .5σ to .9σ — no facilitation effective in less time, none in more — or about duration of a spike potential.

~~Excitatory process at knob not longer than .23σ for full value; anti dromic effect (refractory state of moto neuron) .23σ, upper limit.~~
