# Supplementary material for: The influence of Cajal and Lorente de Nó on Donald O. Hebb’s neuropsychological theory
Source: Front Neuroanat. 2026 Jul 27;20:1861011. doi: 10.3389/fnana.2026.1861011 (PMC13454044; doi:10.3389/fnana.2026.1861011)
Supplement: Supplementary file 2 [file Supplementary_file_2.pdf]

The influence of Cajal and Lorente de Nó on Donald O. Hebb's neuropsychological theory

Richard E. Brown

Appendix 2.

Hebb (1944b) Concept Speculations

April June '44

4 concept speculations - outline for 3rd presentation, following 5(g), with preliminary references to parts already dealtwith.

(4)

(A) prepared to accept - even welcome - innate organization such as Dr Lashley has suggested, with same tissue activated at 18 or thereabouts and higher, with different retinal loci, for "elements" such as line, angle, retinal angular distance.

(B) What I mean by <u>organization</u> behaviorally: ready identification or discrimination, ease of recall and accuracy of recall or identification, ease of association; recognition in any circumstances. The rigid + non-flexible habit as evidence of organization achieved c̄ difficulty, hence not highest level of organization and physiologically: assumption of need of number of parallel LNCs for stable response or perception, possibility of good organization by temporary facilitations, possibility of good organization by innate physiological and anatomical factors, possibility of organization by structural modifications; effect of brain operations on easy vs. difficult discriminations, learning.

Concept of <u>set</u>: idea that organization first built on existing LNS activity - recognition dependent on situation in broad sense, and if in learning any particular sequence of macular analyses was set up, on this also presumably; but with repetitions, likely that order not particularly important, and familiar object would be recognized from any angle, in any (0) setting: this would be a higher or more complete organization.

Idea that space perception fundamental in learning: evidence from rat, <u>very brief</u>, and even in chimp, from his greater ease of associating place and holding it in mind (delayed response evidence), or man (number ~~xxxxxxx~~ forms, etc.: not need to be conscious even if all have them: if numbers learned on space coordinates, superimposed LNC activity would modify the total enough so that we might suppose it only by accident that one would be conscious of space-coordinate basis of identification of numbers, etc.)

Sensory association, the concept as not a function of any one mode or presentation, Lashley's idea that rat learning in maze is not function of any one sense mode;

Similarity, briefly, of concept and percept in man, and relation of this to the theory here developed; introducing the idea of central facilitation producing something that is not an image, intrespectively described, but functions as immage might be supposed to: <u>keeping in mind</u> that the place of its action (particularly in the rat) would be primarily on the non-topological suprasensory LNS of space perception, and tending to produce eyemovement and bodily orientation toward the part of the field whose supra-17 LNS was activated centrally.

5(h) cont'd Idea of autonomous central LNSs, (ACP = aut central pool) with sensory component of organization, but presumably subject to their own physiological and anatomical determinants: normally, presumably, subject to sensory activation, but also capable of autonomous activity; capable of initiating motor activity, but normally capable of activity with only embryonic motor expression;

i. This the system which underlies central determination of the

5 concept speculations

figure, capable of setting figure boundaries where no boundaries are, accounting for the role of experience in determining what is attended to, and for the significance of implicit motor activity (especially eye-movement, in the present theory) - since they would have the same central-initiating potentiality as proprioception would have though not perhaps to the same degree - with an easy discrimination, implicit movement could do the trick; with a hard one, we would actually have to say the word, or make the eye-movement.

Note gestalt failure to deal with the importance of experience in determining the figure - [i.e. in] the factor of "attention".

6. Mechanism of learning

a. Learning already referred to, here deal with learning of actual habits, instead of perceptual learning: return to assumption (6) - facilitation A, transient, facilitation B more stable; possibility of one-trial learning if A lasts long enough to establish B (about whose time requirements we know nothing.)

b. I am deliberately vague about when or where one LNC will (A) facilitate another, and hence make possible structural (B) facilitation; simultaneous activity not enough, because of (1) anatomic separations, (2) apparent fact that one system can inhibit or extinguish another (Dusser de Barenne & McCulloch), (3) factors of timing utilized by Lashley in his theory of repetitive circuits. Idea of diffuse engram makes possible unimportance of anatomicalfactors, but facts of aphasia, selective effects of different locus injury in man and animalsuggests difference, grossly determined, in locus of various LNSs, so this presumably operates.

c. Conception of operation of attention in learning, of operation of ACP in establishing first learning, of weaning of easy response from ACP, or no weaning when complex enough that most available circuits needed for response:

Conception of difficult response always needing ACP to full extent, and phenomenon of learning which is clearly established to well above chance level and yet not perfect; converse phenomenon of learning slow to establish and yet independent of ACP, so attention not needed for reading aloud (e.g.)

Idea that organization of concepts may need ACP, then 2 such concepts not readily associable - [illegible] variable & chief [illegible]

d. ACP on the whole may be capable in man of reproducing stimulus activities well enough for some integrated activity, but this is limited as we know, since paper and pencil or actual manipulation is frequently necessary for effective problem solving; also, is capable of evoking integrated action but normally one may say that inhibitory sets may prevent its actively leading to complete motor action.

e. Before going farther, look at mechanisms of rat learning and law of effect: instead of the maze and the discrimination apparatus, certain peculiarities of behavior may first be attended to as best indication of nature of cerebral organization.

6(e) cont'd

Take first the conception of central control of figure, idea that a part of the field or a pattern in the field may be determined centrally. It has already been assumed that learning of place, direction, fundamental to the rat. Take open field experiment, bare table with only food dish on it: rat will (1) go to a certain place from any position on the table (2) will hunt in that place for food when food moved: place per se recognized. Indicates, in terms of present ideas, central activation of certain place LCS, & what rat does is to orient body to that place & then progress.

we are at once over the difficulty of variable motor response with same effect: by perceptual control. It is assumed that the place-and-direction LCSs are so organized that rat gets cue for turning around when pointed in thewrong direction from actual presentation of wrong side of room (no window, eg.); his frequent orientational examinations of whole set-up in a new position, or with any important change in the position, but <u>not</u> when he is put in a new position in a familiar situation.

f. Next for his ideas on route to food: open rectangle exp's, box-with-tunnel entrance exps, L-tunnel exp's: interpret as meaning perceptual accentuation by ACP of otherwise undifferentiated part of field, as route; from tunnel entrance exps, that this is primarily as a whole, cues as exact markers of part of route come later; from rectangle exps, that no very exact estimate of distance-out-of-sight are possible, so short or long run after crossing barrier does not greatly modify ACP activity, hence route, xx also that route is seen as crossing a certain part of undifferentiated barrier, as above; but L-tunnel exps, that a definite increase in length and change of direction together can do so;

Again primacy of direction, but evidence of summation of perception also, in jumping-stand facts with change of direction. Comparable to rat error here is perhaps our judgments of emotio[n] with set (previous effects of experience) playing an important role without being aware of it, thinking all is given here and now.

g. Now for law of effect~~, and maze learning~~. (1) LCS for training environment, with sub-LCSs for parts of environment and for feeding: (2) this superimposed on previous LCSs of environmental perception; (3) each one so organized that not dependent on visio[n] from one aspect; (4) mechanism of perceptual accentuation, operating perhaps haphazard, perhaps partly determined by earlier food-environment relations; (5) hunger-feeding LCSx first established fxxx, as subordinate to hunger-feeding-environment LCS - animal "expects" or starts looking for food when put into experimental situation; xxxhx (6) as he moves about, partly under control of (4), various place (environmental sub-) LCSs are successively activated (note that (1) is this); (7) in the case of one, however, xxx sensory activation is accompanied by sensory activation of hunger-feeding LCS, and two fuse; (8)

6(g) cont'd

on putting into test situation next time, therefore, hunger-feeding-environment LCS activated, but this now associated with sub-LCS of one particular place, a perceptual accentuation of the part of the field with food in it, hence (9) head and eye turning toward the goal, and progression.

xx Note that this means learning strictly in terms of the "ideas" current at the moment, in terms of the set, of the concepts already developed and of which the animal is capable; it does not mean that any chance series of actions would tend to be fixed, or that all stimuli associated with the goal -physically associated - will become cues to food. But no matter in what part of field rat is put, provided he has been there before during preliminary exploration or provided the relation of that part of the field to the rest is one that has been experienced before (operation of LCS of environment upon which experience of field actually tested in is superimposed), this mechanism would imply an immediate adjustment of response mechanisms to run to food.

h. In maze: speculative of course, but shows how idea of representative or conceptual activity changes problem of elimination of errors or of law of effect.

Assume (1) schema of food place inrelation to schema of environment, (2) representational schema of route - if my introspection means anything, not complete at any one point, but detailed for that point and capable of evoking subsequent detail later but tending to be organized in an experienced sequence (3) on basis of experience, this schema fluctuating (a function of earlier established LCSs): then

rat arrives at A, route schema let us say occurs roughly A-F; rat meets barrier or anticipates it, schema

+ Fernald's exp.

8

6 con'td

by accentuation of B or C alternately, fluctuates - rat chooses B, bumps into dead end, activates schema of dead end, B & dead-end schema fuse, dead end schema encloses concept of reversal, B accentuation at point A means: B, reversal;

alternately, A - LNS of B - LNS of A, accentuation of C, followed by continued progress

alternately, accentuation of route schema diagrammed on maze.

i. More elaborate motor controls, as in man, again a function of perception: obvious fact that the same situation does not evoke different responses, unless the perceived ~~pxrkxxx~~ body is excluded: Assume that man has representative functions, pictures hand in certain place, perceives it in another place: control is certainly complex, but ~~xxxxxxingx~~ not out of order to ~~pxrxxive~~ assume that a number of such relations are learned; also that a number of complex patterns of motion, such as the direction of movement of a screw, are learned with an intrinsice perceptual control: the perceptual equivalences then will provide to an ~~xxtxx~~ great extent for motor equivalences.

That is, we learn not to move certain muscles, in learning a skilled act, but to satisfy certain perceptions; and the combination of perceived situation and conceived situation (imagined outcome) dominates the activity. When we break the motor control down into (1) perception of actual situation (2) imagined situation (3) separation of timing systems from those determining actual effectors used, the problem if not exactly solved is greatly altered, and main difficulty in complex human behavior is lack of experimental data, - I rest my case on the rat.

j. Establishment of a learned response, as consistent and predictable, presumably involves the activation of a minimal number of LNCs; early in learning, before much structural change, a larger number necessary than afterward; the facts of attention seem to suggest that there is a ~~supernumerary~~ central pool of LNCs which can selectively facilitate sensory activities (ACT as already urged) and also that the pool is essential to a new complex learned act; if barely within the subject's pwers, always necessary; with time, and a relatively easy act, not necessary, and is "freed" from the act. The mechanism of freeing I think is found in the involvement of the same pool in new acts, which set up new relationships within it. The pool is, I think, the basis of voluntary action as well as of thought.

k. If you can overlook a reference to the problem of consciousness, think for a moment of what we consider to be involved in human consciousness: if no memory, conscious? clearly not - consciousness therefore implies the capacity to bring up the past at will, to respond to symbolic cues, and so on: I doubt then whether the rat can. It seems to me that the difficulty with delayed response is a lack of clear concepts under central control, and the ability to maintain them clearly -

over
