# Supplementary material for: The influence of Cajal and Lorente de Nó on Donald O. Hebb’s neuropsychological theory
Source: Front Neuroanat. 2026 Jul 27;20:1861011. doi: 10.3389/fnana.2026.1861011 (PMC13454044; doi:10.3389/fnana.2026.1861011)
Supplement: Supplementary file 3 [file Supplementary_file_3.pdf]

The influence of Cajal and Lorente de Nó on Donald O. Hebb's neuropsychological theory

Richard E. Brown

Appendix 3.

Hebb (1944c) Correspondence with Lorente de Nó. 1944-1949

April 28, 1944

Dr. Rafael Lorente de Nó
The Rockefeller Institute for Medical Research
66th Street and York Avenue
New York City

Dear Dr. Lorente de Nó:

The conditions of appointment at this Laboratory provide for periods of study at University or research centers in the North, and I am writing to find out whether you would consent to my coming into your laboratory, for perhaps a month, at some time during the coming summer or autumn.

My object would be to become more familiar with your work, and with the kind of evidence with which your work deals. I am a psychologist, not a neurophysiologist, and would not propose to undertake any research; on the other hand, if I could be useful in your laboratory in any way I should be glad to do so.

I am of course acquainted with a number of your published papers, and it is because of the importance of your research for psychological problems that I should like to know it better. I find that there are limits to what I can get through reading, and in the field of neurophysiology in general my ignorance of technique offers a real barrier to understanding much published work. What I should like to do, therefore, is to bring some incompleted writing at which I could spend any spare time, and to spend as much time as possible learning something about your present work and methods.

You may care to know something about my background and training before coming to a decision. I obtained the Ph.D. degree in physiological psychology with Professor Lashley at Harvard in 1936, and spent a further year with him as research assistant; from 1937 to 1939 I held a Rockefeller research appointment with Professor Wilder Penfield at the Montreal Neurological Institute, studying the effect of brain operation and pathological conditions upon human intelligence. From 1939 to 1942 I taught at Queen's University, Kingston, Ontario. In 1942 I received an appointment here, where I am working at the problem of the effect of cortical extirpations upon behavior in chimpanzee subjects, in collaboration with Dr. Lashley.

There might be a number of reasons why the arrangement proposed would not be feasible, and if so please do not hesitate to tell me. Otherwise I should like to know if there is some particular time which would be more suitable than another, if you are kind enough to consent to such an arrangement.

Yours truly,

DOH:h

D. O. Hebb

THE ROCKEFELLER INSTITUTE
FOR MEDICAL RESEARCH
66TH STREET AND YORK AVENUE
NEW YORK
21

May 1, 1944

Dr. D. O. Hebb,
Yerkes Laboratories of Primate Biology,
Orange Park, Florida

Dear Dr. Hebb:

For the past five or six years I have been working exclusively on nerve physiology. After having acquired some knowledge of the properties of the synapse, I felt the need of carrying out a detailed study of the nature of the nerve impulse.

At present, my work is concerned with the relationship between the production of the nerve impulse and the metabolism of nerve, a problem that perhaps is of little immediate interest to a psychologist. My plan, however, is to eventually return to the study of the central nervous system. This would perhaps offer a better opportunity for your working here.

Will you inform me regarding your plan of work and your training in general physiology and chemistry?

Sincerely yours,

R Lorente de Nó

R. Lorente de Nó

RLN:LS

May 4, 1944

Dr. R. Lorente de No
The Rockefeller Institute for
Medical Research
66th Street and York Avenue
New York, 21

Dear Dr. Lorente de No:

As you perceived, it was your work on the synapse of which I was thinking in writing to you. Your suggestion that another time might be more suitable for a visit to your laboratory seems wise, therefore, and I shall make other plans.

I should not like you to think this was a proposal, by an inadequately trained person, to undertake neurophysiological work for which he was not fitted. Since I hope some time to have the benefit of discussing certain problems of psychological theory with you, I should like to make this clear. If this laboratory were part of one of the large research institutions of the country, a formal visit to another laboratory would not be necessary in order to maintain contact with research in other fields; but here, with a small, predominantly psychological staff, one tends too much to get into a rut in one's thinking. Also, I am profoundly convinced that psychological progress will depend on research such as yours - not that psychologists should necessarily do such research, for there are problems at the level of gross behavior which apparently must be studied as such, but that psychological theory can go no farther without a more detailed basis in neurophysiological fact.

Therefore I had it in mind to go North to work on the preparation of several incompleted papers, while having the opportunity to talk occasionally to persons whose ideas will be affecting psychology ~~in the~~ tomorrow. Such ideas of self-education may appear peculiar, but I shall hope to explain them better when I have an opportunity of meeting you at some future time. Thank you for writing so promptly.

Yours truly,

DOH:h

D. O. Hebb

April 26, 1949.

Dr. Lorente de No,
The Rockefeller Institute for Medical Research,
66th Street and York Avenue,
New York 21, N. Y.

Dear Dr. Lorente de No:

Some time ago you were kind enough to give me permission to use Figure 3 from your paper "Synaptic stimulation of motoneurons as a local process, "Journal of Neurophysiology, 1938, vol. 1, p. 200. The book is now being published by Wiley & Sons, New York, instead of Thomas, which I assume will suit you just as well. I am writing now to ask whether you have still the original drawing and would allow Wiley and Sons to photograph it; or if not, whether I could have it re-drawn by an artist here, and sent to you for approval? I am not sure that this will be necessary: a black and white sketch of the relationships you have shown might be sufficient, but on the other hand your original drawings would be much more effective. As I said in my earlier letter, I believe that my book will be able to show that modern ideas in neurophysiology, and particularly some of those that you have developed, have a revolutionary significance for psychological theory. Consequently, I should like an illustration of yours to be effectively presented in the book.

Yours truly,

D. O. Hebb,
Department of Psychology.

DOH/MH
