# Supplementary material for: The influence of Cajal and Lorente de Nó on Donald O. Hebb’s neuropsychological theory
Source: Front Neuroanat. 2026 Jul 27;20:1861011. doi: 10.3389/fnana.2026.1861011 (PMC13454044; doi:10.3389/fnana.2026.1861011)
Supplement: Supplementary file 4 [file Supplementary_file_4.pdf]

The influence of Cajal and Lorente de Nó on Donald O. Hebb's neuropsychological theory

Richard E. Brown

Appendix 4.

Hebb (1945a) Structure and Origin of Concept Speculations

Written Spring '45

Structure and origin of concept speculation

LNC

A. Antecedent basic ideas:-

1. That new learning is in terms of old learning, grafted on to and not replacing it; existing theory implies that new learning is an establishment of new connections for sensory elements: fact of grafting acknowledged by McGeoch, Woodworth, but they could not deal with it theoretically so it remained as academic point only.

2. That there are standing cerebral activities for which certain sensory processes are necessary — cf "reaction to deficit," emotion in man (+ chimp): ACA = autonomous central action; that normal thought would not be possible in the absence of afferent activity.

3. That vestigial motor "expression" of ACA is more significant than opponents of motor thought (with whom I agree primarily) have realized; indicating a short-circuited motor integration + cf evidence of play, to effect that eventual motor expression is inevitable.

4. That ACA must be considered the direct determinant of behavior; that ACA events are a joint function of afferent activity + preceding ACA activity (influence of ACA on perception, also, was however seen only after system began to be developed).

5. From "early-late" data, that lasting changes must occur in ACA, affecting "intelligence" — no way in which they could be thought of as S-R modifications.

6. That perception is certainly not as anatomically unified as it appears to be — not until I developed this idea did I realize the force of KSL's paper on "functional determinants" (idea developed further into a denial of possibility of an isomorphic level only with development of system).

7. A profound conviction that sensory learning must be provided for. — little more, till ideas developed

8. That very simple perceptions, to us, may have involved a far more complex learning process than one would think: Nissen's evidence; Senden; "early-late" evidence; this, particularly in conjunction with (1), bears on (5).

B. Ideas primarily developed in looking for physiological solution.

1. Successive part reinforcement in perception.

2. Specific conception of short-circuit motor integration of perception; supporting evidence in subjective observation, + in nystagmus.

3. Dual trace mechanism.

4. No intelligible basis for anatomically unified isomorphic area corresponding to "conscious" perception – no possible topographic correspondence – hence away from this idea completely to (6) below

5. Arising to a great extent from (A 7): the idea that KSL's step-wise development of abstraction may apply to Δ, O, etc.; and that the divergences of the rat's generalization may be more significant than at first appears; and that actually KSL's data do not support the idea that injuries outside 17 have no effect on discrimination of visual objects, nor that lesions inside 17 have an all-or-none effect for pattern vision.

6. Diffusity of perceptual + intellectual activity – implied possibility of multiple parallel (functionally parallel) fibres with a common effect – + physiologically unified – This providing, unexpectedly, the explanation of the failure of cerebral lesions in association areas to produce specific, restricted defect.

C. Positive contributions from systematization

1. Suggested mode of influence of ACA upon process of perception

2. Suggested reason why "set" + so on are so effective in learning — unification of (1) set-recognition problem and (2) set-learning problem, + explanation: ACA-perception relation =

3. Idea from (2) of accentuation of part of field — not an image, but a process half way between image and sensation, + brand-new in suggesting a physiological basis without any reference to consciousness. — see (9) below

4. Idea of motor equivalences as same problem as "reaction to deficit" etc.; ~~[illegible]~~ ~~[illegible]~~ — an important synthesis, though obvious once stated; and thus an explanation of the motor equivalences. ~~law of effect, elimination of errors.~~

(but ~~[illegible]~~, KSL has already done it)

5. See B(6).

6. That perceptual learning, and sensory association of organized percept, ~~with~~ organized percept, is physiologically comprehensible — + see what this does to the law of effect, + the problem of the elimination of errors!

If all this seems obvious, I say it is not; and for evidence I point to the literature concerning ① law of effect, and ② elimination of errors = just look it over!

In other words, to say that these ideas are "just" translation, or means nothing — look at the effect of translation, or rather, what the lack of it has done to the literature:

C cont'd

"Lorente de Nó circuit"

7 Hypnagogic imagery as persistent activity of higher (perceptual level = supra-17) LNC activity; lack of precision due to greater activation of some systems than others, or lack of others ("functional determinants" idea of multiple anatomical basis, physiologic unity, applied to perception); and the general idea that generalization is immanent in perception, the very fact of discrete (anatomically) systems being involved providing basis of generalization — inevitable, if this is so.

8. Ideas of timing of motor sequences, errors of speech, due to organization of motor ~~behavior~~ activity by simultaneous "functional determinants" systems.

9. Any preceding attempts to deal with problem of perception have assumed its topographical unity and approach to geographic features of stimulus — + no way to allow for ACA — perception effect — or ~~ACA~~ ACA effect on fig.-gd division — while the present system makes it obvious.
