# Supplementary material for: The influence of Cajal and Lorente de Nó on Donald O. Hebb’s neuropsychological theory
Source: Front Neuroanat. 2026 Jul 27;20:1861011. doi: 10.3389/fnana.2026.1861011 (PMC13454044; doi:10.3389/fnana.2026.1861011)
Supplement: Supplementary file 5 [file Supplementary_file_5.pdf]

The influence of Cajal and Lorente de Nó on Donald O. Hebb's neuropsychological theory

Richard E. Brown

Appendix 5.

Hebb (1945b) Precis: The Structure of a Set of Neuropsychological Speculations

Precis: The Structure of a Set of Neuropsychological Speculations (March - July 1945)

Outline. The introduction (A) deals with aim, scope and mode of approach, from the point of view of a possible writing up and presents a fundamental assumption concerning the nature of cerebral action.

Part I. (B) to (F) tries to establish the probable truth of a series of propositions concerning perception. (G) then makes a bald assumption concerning the nature of learning, and (H) tries to show that learning is in terms of previous learning - that new habits "utilize" instead of merely replacing older habits.

Part II. From this point on, the attempt is made to show that ~~certain conclusions follow from B to H (treated as postulates) concerning the~~ a schema of the physiological control of behavior can be developed by utilizing these preliminary ideas, and to develop new conceptions ~~of~~ ~~that~~ ~~~~ (from the schema) (of value in the analysis of real behavior.)

The ~~system~~ schema does not make specific predictions, and thus ~~amounts really to~~ suggests only a reformulation of the problem ~~of the control of behavior~~, instead of providing an explanation. There is an approach to explanation, however, in the fact that it shows how a variety of psychological problems ~~are~~ (may possibly be) unified, and how it ~~becomes~~ is possible for example to conceive of a mechanism of the effect of attention on learning, in physiological terms; ~~or to show~~ and suggests that if the hurdle of perceptual generalization can be got over, the problem of motor generalization and its perceptual control becomes physiologically explicable (in general terms, of course, and for simple cases). The system is not merely a translation into physiological terms, therefore, but does ~~accomplish~~ suggest the basis of a considerable synthesis and makes possible an intelligible formulation of the problems of attention and thought, as a function of central processes.

Proposition G Without any direct experimental evidence to support it, the assumption is made that lasting structural changes occur to reinforce and perpetuate the temporary, dynamic facilitation whose existence is a necessary inference from Lorente de No's work. This makes the assumption that there is a dual mechanism of the trace, a"dynamic" plus a "structural engram".

LSC?

p61 of TOOB org Behav

Discussion:

G 1 Hilgard and Marquis (1940, p. 331) have shown that a dynamic but transient trace-mechanism is an inference from Lorente de No's conclusions (1) that a cell is fired only by two or more simultaneously active fibres and (2) that internuncial fibres are organized in closed, self-exciting chains. They go on to point out that this trace would be unstable and can not explain long-term memory. It is equally important to note, however, that the conception is valuable as an explanation of the memory which seems to be established instantaneously; and the instability of the mechanism is actually an advantage in explaining the memory which seems to be as evanescent as it is immediate. In the repetition

of digits, for example, an interval of a few seconds is enough to prevent any interference effect from one series of digits to the next. We can, therefore, utilize the conception in theorizing if it is possible to provide also for long-term memory.

G 2. The assumption is consequently made that long persistence or repetition of the dynamic facilitation (of (G 1) above) tends to induce structural changes which reinforce the facilitating action. This assumption can be put as follows: when cell A is in propinquity with cell B and repeatedly or persistently takes part in firing B, some growth process or physiological change takes place in one or both cells such that A's efficiency, (as one of the cells) firing B, is increased.

Hebb's synapse

The most obvious suggestion concerning the nature of this change is that it is the development of "synaptic knobs" (not "end-buttons": Lorente de No, 1938a, p. 198). There is certainly no decisive evidence that this is so, and the change if it exists might be metabolic, affecting cellular rhythmicity and limen; or again, both metabolic and structural changes might be involved. In the speculation which follows these initial propositions it may not be important which basis, metabolic or structural, the mechanism of the postulated stable increase of facilitation between cells is assumed to have; but there are a number of facts which make the development of Lorente de No's synaptic knobs the most plausible mechanism, and for the sake of definiteness it is this which is selected as the basis of the postulated stable facilitation accounting for long term memory.

G 3 In this subsection accordingly an attempt is made to show more precisely what is involved in the assumption that actual growth changes occur in learning, to show that the idea is not wholly implausible.

(a) The assumption does not require a prolongation of the axon or axon collaterals, nor any extensive ameboid development of the cell determined by action at a distance. The assumption made is that the structural development occurs only when cell A is already close enough to cell B to fire it. Lorente de No (1938, p. 198) points out that "end-button" is a misleading term, deriving from earlier studies with inadequate staining technique, and that the buttons are actually thickenings of axon fibrils where they pass in proximity to dendrite or soma of a second cell. The thickenings are often, or usually, multiple; Lorente's figure (p. 200) shows for example a single fibril with three knobs on the soma of a motoneuron plus two others on a second cell (not stained). It has been demonstrated that one cell can fire a second with which it has contact only (Arvanitaki, 1942); and the efficiency of the firing would be increased by the closer and enlarged contact produced by the development of the knob, since the whole implication of Lorente de No's work on the synapse is that the greater the region of disturbance in the second cell the greater the probability of ~~trans-~~ arousing an impulse in the ~~mission~~.

(b) The fibre-structures in which activity occurs following gross excitations in the same cortical region are inevitably interlaced and could be expected to converge at many points, and at all such points an interfacilitation could occur without any outgrowth of fibrils or "end-buttons". The assumption which has been made, of structural changes which have a stable facilitating action to reinforce the unstable dynamic facilitation implied by Lorente de No's ideas, does not imply any connection between anatomically discrete regions but only the

accentuation of an anatomical intimacy already existing. (C) Also, the diffusity of cortical conduction is apparently such that two excitations in different cortical areas would often be conducted to a common area. For example, simultaneous visual and auditory excitations may fire into the same association areas. There would be, then, some tendency for visual-auditory associations to occur; but it can also be assumed that the number of points in common would be greater when two excitations originate in the same cortical field, and thus that the establishment of an effective interfacilitation in a single sensory mode would be more probable, and established quicker, than when two separate sensory fields are involved.

G 4 One possible difficulty for these ideas is the necessity for synchronization if one reverberating system is to facilitate another. However, this factor of the periodicity of the cell must be approached statistically, since we are not dealing with single-fibre circuits but with populations of fibres in which there would be a statistical dispersion in time of arrival of impulses at any synapse (and also since there might be a constant background of tonic facilitation in the neuropil of the cortex similar to that of the reticular substance of the brain stem). It can be concluded on anatomical grounds, as in the preceding paragraph, that effective facilitation of one reverberating system or another may be more or less probable, or readily established; and similarly on physiological grounds - that is, given anatomical propinquity, the physiological factor would vary from extreme asynchrony, with no interfacilitation, to complete synchrony at one or more synaptic points. From the considerations presented, we can infer that an integration of two simultaneously persistent or recurrent excitations is always possible; the probability that it

will occur readily in a specific instance cannot of course be stated, but in principle this can be regarded as a statistical matter, with the implication that the probability of association for two particular excitations is to be established experimentally.

The assumption made here is that in general integration is probable, with a range of variation in different instances from no association to one which is readily established and immediate.

G 5 One further feature of such reverberatory systems should be mentioned. An increased facilitation at the various synapses by the postulated growth process, or by the simultaneous activity of a second system, seems necessarily to imply a change of the frequency characteristics of the system. This in turn implies that both fractionation and recruitment would occur, some elements in the system would become ~~less synchronized~~ asynchronous with it and ~~tend to~~ drop out; and ~~[illegible]~~ others, not originally in harmony with the system, would ~~tend to~~ become so, as its frequency characteristics change, and hence ~~to~~ (would) be incorporated in it.

Thus with prolonged activity in a reverberating system, or frequent repetition, these things would tend to occur: (1) a lowering of synaptic thresholds, by the postulated growth process, with an internal increase of physiological unity, (2) an integration with other simultaneously active systems, and (3) a "structural shift", or dropping out of some components and an addition of others, as the result of changes (1) and (2).

G 6 In such a discussion it is hard to know to what extent the inferences which are made are ad hoc with respect to the use later made of them. In general terms, the inferences are defensible, but there may well have been a selection of facts involved. The direction of speculation is good inasmuch as it avoids the rigidity of a fibre-to-fibre connectionism, but it also cannot at present lead to specific, testable predictions. In addition to the unknowns which have already

been mentioned, there is also the unknown physiological action of the suppressor areas of the cortex. It is as well to have these things clearly stated, so that the adequacy of the "explanations" proposed in subsequent discussion will not be over-rated.

G 7 ~~It can be observed, finally, that although in justification of~~ Although the assumption of the establishment of some form of connection between cells ~~that this is an assumption which has been made in the past by~~ a ~~number of writers, physiological as well as psychological. It~~ has been forcefully criticised (by KSL and Gestalt writers) on several grounds, ~~but~~ the present formulation is significantly different from earlier ones and ~~is not~~ open to the same objections. ~~aims to meet the criticisms.~~ (may not be)

(1) The source of the first and main ~~one~~ criticism has already been referred to - the apparent inconsistency between perceptual generalization and the conception of a structural trace (A4) - and will be dealt with in detail in later sections. Other criticisms of the ~~[illegible]~~ hypothesis of cellular connections can be summarized here: (2) that the slowness of growth processes would not account for the rapidity of some learning (a doubtful point, since we know nothing of the time required for ameboid changes in the neuron; but it is at any rate provided for by the dual-trace hypothesis of this section, - the dynamic trace presumably could be instantaneously established); (3) that concurrent neural events actually are very often not associated (e.g., if a sensory event is not attended to, it is not associated with the response that follows it): this point is fully provided for in the schema of learning which is presented later; and (4) Lashley's extirpation experiments, showing that no single small block of cortical tissue is necessary to a learned act. This criticism applies only to a "single-route" theory, and is met by the assumption that the connections involved in a learned response would never be single or concentrated in a small

area, but would be multiple and diffuse. This is actually more than an *ad hoc* assumption; it appears to follow by necessity from the physiologically established facts of the diffusity of cerebral transmission from the sensory areas of the cortex. Besides avoiding a criticism that would otherwise be very serious, the assumption seems to offer a new understanding of the facts of behavior following cerebral lesions (Lashley, 1929, 1942, 1943, 1944; Hebb, 1942).

In general it can be said that the consequences of Lorente de Nó's demonstration that the synapse is crossed only by the simultaneous action of two or more impulses has a much greater significance for psychological theory than appears on the surface. The connections postulated here are between single cells, but the single cell is not the unit of excitation, and it is to a great extent by reason of this fact that the postulate avoids the rigidity of older ideas of "synaptic block" and "route formation".

make explicit about my earlier paper "innate" 1937.
